# A Review of the EUSO-Balloon Pathfinder for the JEM-EUSO Program

**DOI:** 10.1007/s11214-022-00870-x

**Published:** 2022-02-01

**Authors:** J. H. Adams, S. Ahmad, D. Allard, A. Anzalone, S. Bacholle, P. Barrillon, J. Bayer, M. Bertaina, F. Bisconti, C. Blaksley, S. Blin-Bondil, P. Bobík, F. Cafagna, D. Campana, F. Capel, M. Casolino, C. Cassardo, C. Catalano, R. Cremonini, S. Dagoret-Campagne, P. Danto, L. del Peral, C. de la Taille, A. Díaz Damian, M. Dupieux, A. Ebersoldt, T. Ebisuzaki, J. Eser, J. Evrard, F. Fenu, S. Ferrarese, C. Fornaro, M. Fouka, P. Gorodetzky, F. Guarino, A. Guzman, Y. Hachisu, A. Haungs, E. Judd, A. Jung, J. Karczmarczyk, Y. Kawasaki, P. A. Klimov, E. Kuznetsov, S. Mackovjak, M. Manfrin, L. Marcelli, G. Medina-Tanco, K. Mercier, A. Merino, T. Mernik, H. Miyamoto, J. A. Morales de los Ríos, C. Moretto, B. Mot, A. Neronov, H. Ohmori, A. V. Olinto, G. Osteria, B. Panico, E. Parizot, T. Paul, P. Picozza, L. W. Piotrowski, Z. Plebaniak, S. Pliego, P. Prat, G. Prévôt, H. Prieto, M. Putis, J. Rabanal, M. Ricci, J. Rojas, M. D. Rodríguez Frías, G. Roudil, G. Sáez Cano, Z. Sahnoun, N. Sakaki, J. C. Sanchez, A. Santangelo, F. Sarazin, V. Scotti, K. Shinozaki, H. Silva, J. F. Soriano, G. Suino, J. Szabelski, S. Toscano, I. Tabone, Y. Takizawa, P. von Ballmoos, L. Wiencke, M. Wille, M. Zotov

**Affiliations:** 1 University of Alabama in Huntsville, Huntsville, USA; 2 Omega, Ecole Polytechnique, CNRS/IN2P3, Palaiseau, France; 3 APC, Univ. Paris Diderot, CNRS/IN2P3, CEA/Irfu, Obs de Paris, Paris, France; 4 INAF - Istituto di Astrofisica Spaziale e Fisica Cosmica di Palermo, Palermo, Italy; 5 Istituto Nazionale di Fisica Nucleare - Sezione di Catania, Catania, Italy; 6 Université Paris-Saclay, CNRS/IN2P3, IJCLab, Orsay, France; 7 Institute for Astronomy and Astrophysics, University of Tübingen, Tübingen, Germany; 8 Istituto Nazionale di Fisica Nucleare - Sezione di Torino, Torino, Italy; 9 Dipartimento di Fisica, Universitá di Torino, Torino, Italy; 10 Karlsruhe Institute of Technology, Karlsruhe, Germany; 11 Institute of Experimental Physics, Slovak Academy of Sciences, Košice, Slovakia; 12 Istituto Nazionale di Fisica Nucleare - Sezione di Bari, Bari, Italy; 13 Istituto Nazionale di Fisica Nucleare - Sezione di Napoli, Naples, Italy; 14 KTH Royal Institute of Technology, Stockholm, Sweden; 15 RIKEN, 2-1 Hirosawa, Wako, Saitama Japan; 16 Istituto Nazionale di Fisica Nucleare - Sezione di Roma Tor Vergata, Roma, Italy; 17 IRAP, Université de Toulouse, CNRS, Toulouse, France; 18 CNES, 18 avenue Edouard Belin, Toulouse, France; 19 Universidad de Alcalá, Madrid, Spain; 20 Colorado School of Mines, Golden, USA; 21 Uninettuno University, Roma, Italy; 22 Center of Research in Astronomy, Astrophysics, and Geophysics, Algiers, Algeria; 23 Space Sciences Laboratory, University of California, Berkeley, CA USA; 24 National Centre for Nuclear Research, Lodz, Poland; 25 Skobeltsyn Institute of Nuclear Physics, Lomonosov Moscow State University, Moscow, Russia; 26 Universidad Nacional Autónoma de México, Mexico City, Mexico; 27 Universidad de León, León, Spain; 28 ISDC Data Centre for Astrophysics, Versoix, Switzerland; 29 University of Chicago, Chicago, USA; 30 Dipartimento di Scienze Fisiche, Universitá di Napoli Federico II, Naples, Italy; 31 Lehman College, City University of New York, New York, USA; 32 Dipartimento di Fisica, Universitá di Roma Tor Vergata, Roma, Italy; 33 Istituto Nazionale di Fisica Nucleare, Laboratori Nazionali di Frascati, Frascati, Italy; 34 ECAP, University of Erlangen-Nuremberg, Erlangen, Germany

**Keywords:** JEM-EUSO, Ultra-High Energy Cosmic Rays, Extensive air showers, Stratospheric Balloon

## Abstract

*EUSO-Balloon* is a pathfinder for *JEM-EUSO*, the mission concept of a spaceborne observatory which is designed to observe Ultra-High Energy Cosmic Ray (UHECR)-induced Extensive Air Showers (EAS) by detecting their UltraViolet (UV) light tracks “from above.” On August 25, 2014, *EUSO-Balloon* was launched from Timmins Stratospheric Balloon Base (Ontario, Canada) by the balloon division of the French Space Agency CNES. After reaching a floating altitude of 38 km, *EUSO-Balloon* imaged the UV light in the wavelength range ∼290–500 nm for more than 5 hours using the key technologies of *JEM-EUSO*. The flight allowed a good understanding of the performance of the detector to be developed, giving insights into possible improvements to be applied to future missions. A detailed measurement of the photoelectron counts in different atmospheric and ground conditions was achieved. By means of the simulation of the instrument response and by assuming atmospheric models, the absolute intensity of diffuse light was estimated. The instrument detected hundreds of laser tracks with similar characteristics to EASs shot by a helicopter flying underneath. These are the first recorded laser tracks measured from a fluorescence detector looking down on the atmosphere. The reconstruction of the direction of the laser tracks was performed. In this work, a review of the main results obtained by *EUSO-Balloon* is presented as well as implications for future space-based observations of UHECRs.

## Introduction

Several decades after the first report of a particle with energy of 10^20^ eV (Linsley 1963), the origin and nature of Ultra-High Energy Cosmic Rays (UHECRs) is still not clearly understood. This is mostly due to the extremely low particle flux – around 1 particle per km^2^ per century - reaching the Earth at energies on the order of 5×10^19^ eV. Currently, two ground-based observatories, the Telescope Array (TA) (Abbasi et al. 2018) and the Pierre Auger Observatory (PAO) (Aab et al. 2020), are observing the sky from the Northern and Southern hemisphere, respectively. In the future, an important step forward in studying UHECRs could come from space-based experiments which have the potential to look at the whole sky with a much larger instantaneous exposure (Panasyuk et al. 2015). In this respect, *JEM-EUSO* (Joint Experiments Mission: Extreme Universe Space Observatory) (Adams et al. 2015b) is the most advanced program with projects from ground, on stratospheric balloons, and in space to test the feasibility and realize a large-scale mission in space devoted to UHECR science.

*EUSO-Balloon* (Adams et al. 2015a) is a mission within the *JEM-EUSO* program. The main objectives of the *EUSO-Balloon* flight were to perform: a) a full scale end-to-end test of the instrumentation in the *JEM-EUSO* detector and several key technologies used by *JEM-EUSO*; b) a detailed measurement of the photoelectron counts in different atmospheric and ground conditions and its implications for estimates of the exposure of a space-based mission; c) the first measurement of air-shower-like events from the edge of space as a proof of principle for their detection and reconstruction. *EUSO-Balloon* was launched by the French Space Agency CNES from the Timmins base in Ontario, Canada, on the moonless night between August 24th and 25th, 2014. After reaching its floating altitude of about 38 km, *EUSO-Balloon* imaged the UltraViolet (UV) intensity in the wavelength range ∼290–500 nm for more than 5 hours before descending to ground level.

The EUSO-Balloon refractor telescope consists of two Fresnel lenses of ∼1 m^2^ area and the focal surface is filled with Multi-Anode PhotoMultiplier Tubes (MAPMTs). The spatial and temporal resolutions of the detector at floating altitude is ∼130 m and 2.5 μs, respectively. The full field of view (FoV) in nadir mode is about 11 degrees. The UV snapshots collected by the instrument is complemented by infrared images taken by an infrared camera on-board *EUSO-Balloon* (Rodríguez Frías et al. 2015).

During 2.5 hours of the *EUSO-Balloon* flight, a helicopter circled under the balloon to operate UV flashers (Adams et al. 2015c) and a UV laser (Abdellaoui et al. 2018a) to simulate the optical signals from UHECRs, to calibrate the apparatus, and to characterise the optical atmospheric conditions. During the flight, *EUSO-Balloon* took more than 30 million images that were analysed to extract different information: performance of the different parts of the detector; response of the detector to the UV flashers and laser events; UV radiance from the atmosphere and from the ground in different conditions (e.g. clear and cloudy atmosphere, forests and lakes, city lights).

In this work, a review of the main results obtained by *EUSO-Balloon* is presented as well as implications for future space-based observations of UHECRs. The paper summarizes already published works on specific topics, which are here presented in an organic way to provide an overview of the objectives and results of the *EUSO-Balloon* mission. A summary of the paper content and of the most relevant published works is as follows. Section 2 outlines the *EUSO-Balloon* instrument in all its major components (Adams et al. 2015a) while Sect. 3 describes the *EUSO-Balloon* flight and the role of the helicopter underflight, and ends by reporting on the inflight performance of the instrument (Abdellaoui et al. 2018a, 2017). Section 4 summarizes the scientific results of the flight with particular attention to the estimation of the UV intensity in different locations and atmospheric conditions (Abdellaoui et al. 2019; Mackovjak et al. 2015), as well as to the retrieval of the cloud-tops (Tabone et al. 2015; Merino et al. 2015), and to the reconstruction of the laser shots and the imaging perfomance (Abdellaoui et al. 2018a). This section ends by describing examples of unidentified events found in post-flight analysis (Jung 2017). The conclusions and the perspectives for the future missions of the *JEM-EUSO* program are outlined in Sect. 5.

## The EUSO-Balloon Instrument

**Fig. 1:**
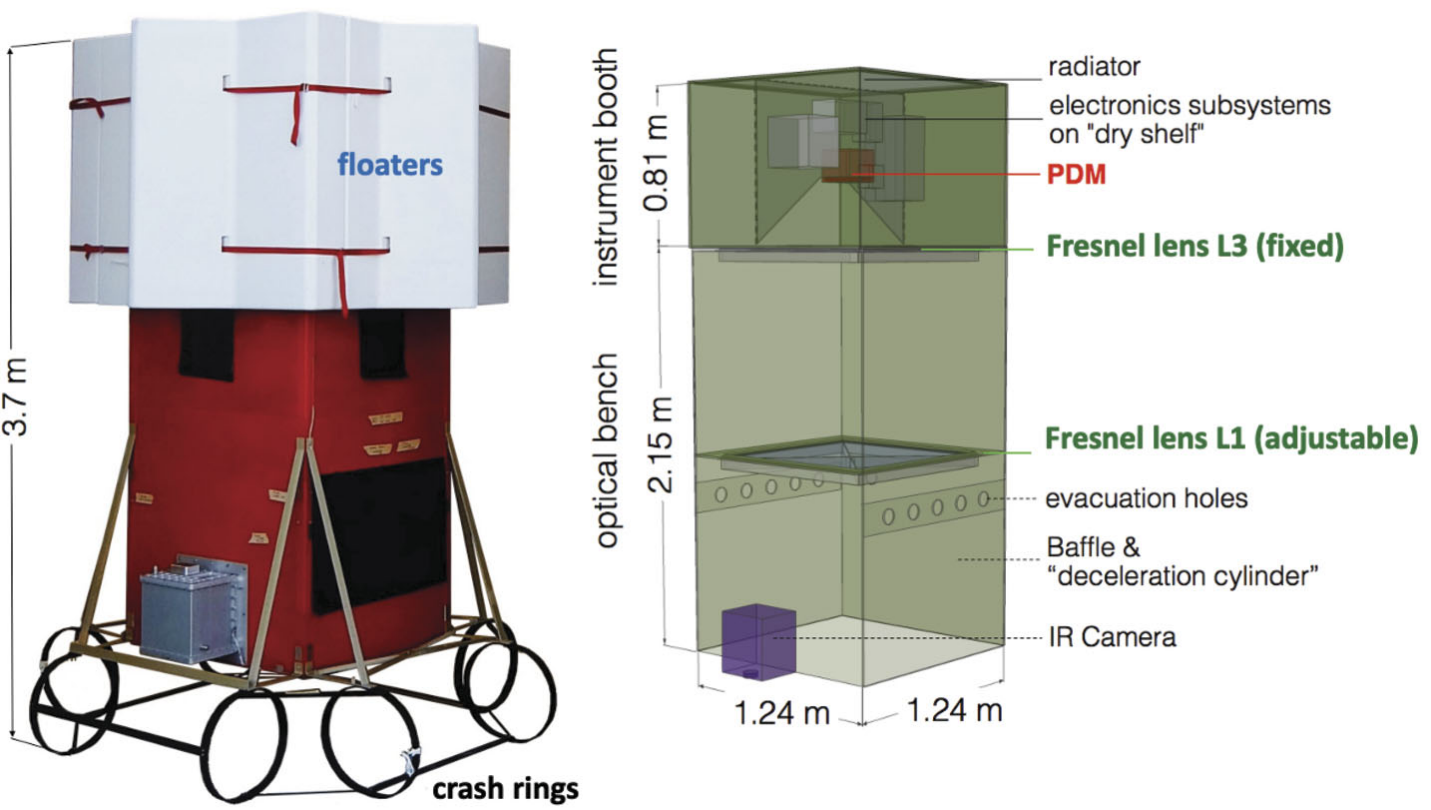
Left: photo of *EUSO-Balloon*, ready for its first flight from Timmins, Ontario, Canada, August 2014; right: schematic view of the instrument booth and optical bench, without floaters and “crash rings”

**Fig. 2:**
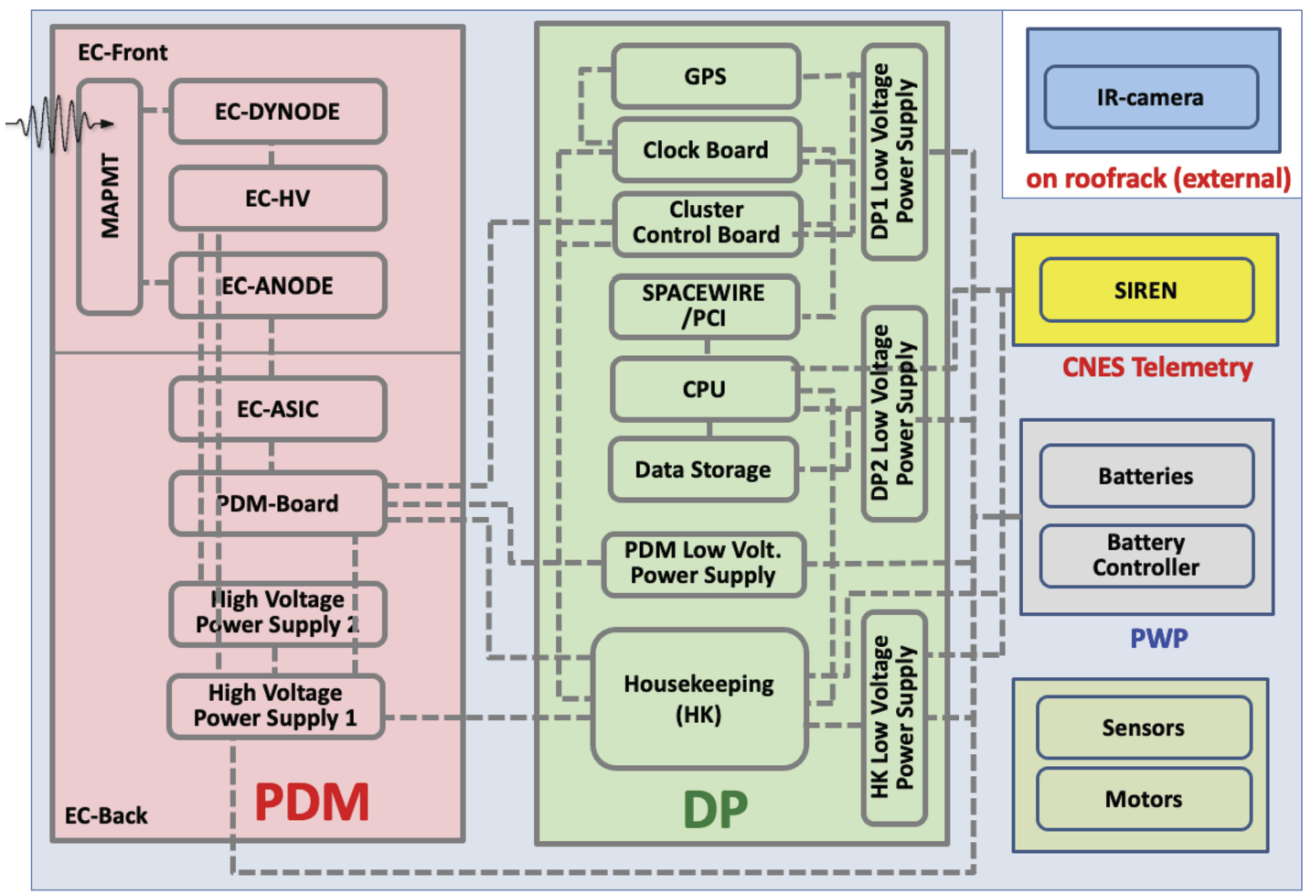
Functional block diagram of the *EUSO-Balloon* electronics with the Photo-Detector Module (PDM), the Data Processor (DP) and the Power components (PWP)

A global view of the *EUSO-Balloon* instrument is shown in Fig. 1 – its two main components being the *optical bench* and the *instrument booth*. The main driver for determining the general layout came from optical specifications, i.e. the pixel size and the FoV which had to be representative for the UV intensity within the pixel of a standard *JEM-EUSO* Photo-Detector Module (PDM). An electronic block diagram of the instrument, summarizing the various subsystems and components is shown in Fig. 2. Besides the focal surface detector (PDM) and associated electronics (Data Processor, DP), which are described below, the instrument booth houses the telemetry system (SIREN), CNES specific instrumentation (Flight Chain Instrumentation, Hub), and two battery-packs.

The development of the components and sub-assemblies is based on similar *JEM-EUSO* components and sub-assemblies, which have all been designed and built within the *JEM-EUSO* collaboration. *EUSO-Balloon* components were used to instrument the *EUSO-TA* telescope deployed in Utah (Abdellaoui et al. 2018b) during 2013-2015 and the balloon-borne *EUSO-SPB1* telescope (Wiencke and Olinto 2017) that flew for 12 days in 2017. Upgrades of these versions are flying on the International Space Station (ISS) as components of the *Mini-EUSO* mission (Bacholle et al. 2021) since 2019. At the time of the *EUSO-Balloon* flight in August 2014, all components had undergone successful thermal-vacuum tests but were not entirely space-qualified yet.

### The Photo-Detector Module (PDM)

**Fig. 3:**
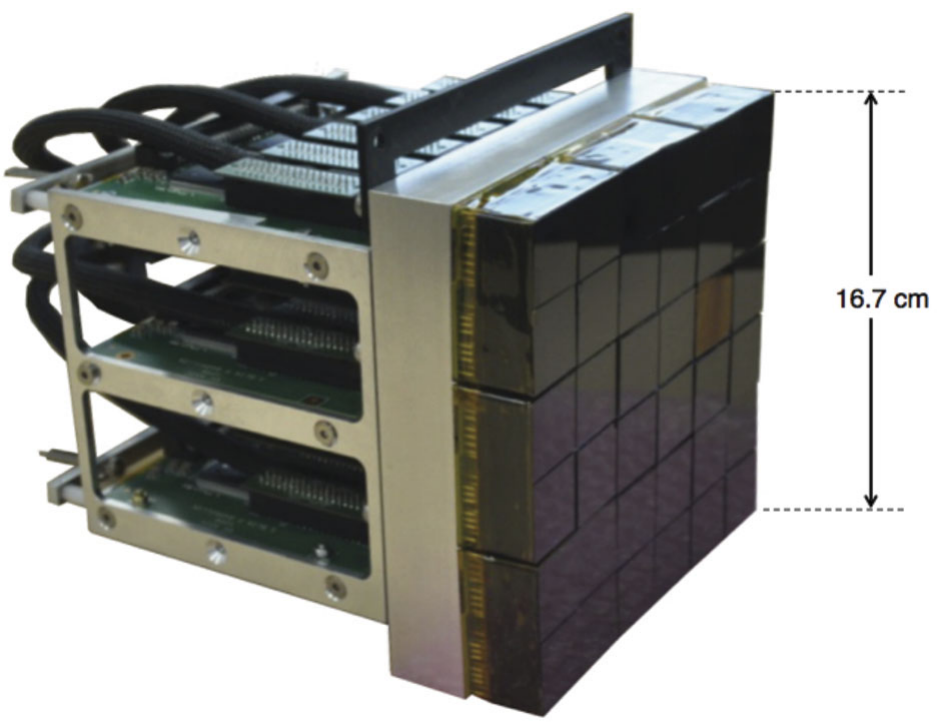
The PDM: the 36 MAPMTs are covered with UV band-pass filters; four MAPMTs form an EC. The PDM includes 9 ECs, 6 EC-ASIC boards, and a PDM board. Each layer on the back hosts two EC-ASIC boards. The EC units are already potted in the present figure (see text for details)

The UV light collected by the telescope is focused onto the PDM (Fig. 3), which is composed of 36 MAPMTs, associated front-end electronics, high-voltage power supplies, and trigger logic. The key characteristics of the focal surface of this UV camera is its spatial resolution of a few mm, its double-pulse resolution of a few nanoseconds and a quantum efficiency of up to 30%, allowing for the detection of single photons. As the focal plane of *JEM-EUSO* is expected to consist of ∼140 closely packed PDMs, a four-side buttable design is implemented on *EUSO-Balloon*: it consists of a compact array of $6\times 6$ MAPMT covering a detection surface of 16.7 cm × 16.7 cm, and it is fully contained in a volume of 17 cm × 17 cm × 20 cm with its mechanical support and front-end electronics included.

For the various tasks of photodetection, analog processing, and digital processing, the PDM is composed of the following components and subcomponents:

- 36 MAPMTs organized in 9 Elementary Cell (EC-units), consisting of $2\times 2$ MAPMTs each,
- 6 EC ASIC boards for their readout (referred to as the EC-ASIC, each EC ASIC board is associated to 6 MAPMTs belonging to 3 different EC-units for mechanical reasons),
- the High-Voltage Power Supply (HVPS)
- the PDM board which includes a Field Programmable Gate Array (FPGA).

Every MAPMT (Hamamatsu R11265-103-M64) has 8 × 8 pixels of about 2.9 mm × 2.9 mm each, and a global footprint of 26.2 mm × 26.2 mm. A UV transmitting filter is bonded to the window of the MAPMT with optical glue. The filter (a SCHOTT BG3 with anti-reflection coating) transmits UV light in a band between ∼290 and 500 nm. The MAPMTs needs 14 different high voltage lines, the highest potential being delivered to the photocathode, the others to the 12 dynodes and the grid. During the flight the photocathode voltage was set to 950 V which corresponds to a gain of $1.1 \times 10^{6}$.

The EC-units are fixed individually to the PDM mechanical frame. The ECs are slightly inclined up to 2.48 deg to approximately follow the aspherical geometry of the focal surface of the optics. The whole EC-Unit block ($2 \times 2$ MAPMTs) is potted to prevent high voltage sparking at the low-pressure conditions present in the stratosphere as the photo-catode voltage is set at −950 V while the metallic frames around is set at ground level. At the rear of the MAPMT array, 6 EC-ASIC boards are stacked in parallel inside a mechanical frame with their plane perpendicular to the photocathodes’ surfaces. Behind the EC-ASIC stack, the PDM board is fixed with its plane in parallel with the focal surface. The HVPS comes in four boxes. Three are used to hold nine independent HV generators. The fourth containes the control for the switches.

The EC-ASIC provides the front-end electronics that performs the MAPMT full-time analog readout. It detects and counts individual photons in each channel. The counts are summed over time interval called Gate Time Units (GTUs). Each GTU lasts 2.5 μs, however, the active portion of the GTU, when analog to digital conversion is performed, is limited to 2.3 μs. The core of the EC-ASIC is the ASIC named SPACIROC V1 (Spatial Photomultiplier Array Counting and Integrating ReadOut Chip, see Miyamoto et al. 2013). SPACIROC V1 is capable of processing in parallel all the anodes of a single MAPMT. In SPACIROC V1, the two pulse separation is 30 ns which limits the maximum number of counts per channel to ∼30 per GTU taking into account the random arrival times of the photons. In parallel to the 64 photoelectron counting channels, a signal integration is done for each GTU on the anode current sum in groups of 8 adjacent pixels so as to correct for counting non-linearity and saturation. Each double-sided EC-ASIC board hosts 6 ASICs, three per face.

The HVPS can operate the MAPMTs of an EC-Unit either in the standard photon counting mode with high gain or in a reduced gain mode in order to avoid damage from an intense light pulse extending over milliseconds or even more. The HVPS consists of a miniaturized Cockroft-Walton generator (Bacholle et al. 2015a), which delivers 14 different high voltages, a switch system used to reduce the photocathode voltage to lower the MAPMT collection efficiency, and the logic system to manage the interfaces with the other elements of the electronic chain. The HVPS boxes are potted to prevent coronal discharges. The advantage of the Cockroft-Walton generator is its low power consumption, a desirable feature in a battery-powered system. The HVPS is stable over a range of input battery voltages.

The PDM uses a Virtex6 XC6VLX240T FPGA to handle the interface with the Data Processor (DP), specifically the HouseKeeping (HK) and the Cluster Control Board (CCB). The PDM board also manages the configuration and the 1 GB/second data stream from the 6 EC-ASIC boards. It transmits a part of the data to the CCB in the DP. During the flight, data transmission was controlled by the CCB, based on a CPU request. Various parameters of the PDM’s response such as the sensitivity and detection performance as well as absolute calibration of the PDM are treated in (Dagoret-Campagne et al. 2015) and (Moretto et al. 2015).

### The Data Processor

The different sub-assemblies of the Data Processor (DP) collect and process the data from the PDM and also include the housekeeping system. It handles the on-board storage and sends a select subset of the data to the telemetry system. The DP is contained in an aluminum crate to assure thermal contact with the radiator; it is composed of

- Cluster Control Board (CBB),
- CPU,
- Data storage (DST),
- HouseKeeping (HK),
- Clock Board (CLKB),
- GPS Receiver (GPSR),
- Data Processor Power Supply (DP-LVPS).

The CCB (Cluster Control Board) is developed around a Xilinx Virtex-4 FX-60 FPGA. It collects data from the PDM board, processes and classifies the received data. The CCB has a second level trigger for additional filtering as explained later. The CLKB hosts the interface with the GPSR. It tags the events with their arrival time (UTC) and geographic location (both provided by GPSR). It also measures the up-time and dead-time of the instrument and provides signals for time synchronization of the event. Most of the functions of the CLKB are implemented in a Xilinx Virtex-5 XC5VLX50T FPGA.

The CPU, based on an Atom N270 1.6 GHz processor, collects data from the CCB and CLKB through two (200 Mbits/sec) SpaceWire links. It manages the data storage and handles the interface with the telecommand/telemetry system. One acquired event represents roughly 330 kB of data. Since only a limited data rate can be transmitted to the ground through CNES’ NOSYCA telemetry system, all data are stored on-board. The mass storage is composed of two Solid-State Drives (SSD), each one with 512 GB capacity operating in fault-tolerant mode RAID 1 (Redundant Array of Independent Disks). The HK system collects telemetry from several sub-systems of the instrument in slow control mode. It is responsible for monitoring voltages and currents of the Low Voltage Power Supply (LVPS), and has a serial bus to convey telemetry and telecommands through the CPU interface and to other sub-systems. The HK system is implemented around an off-the-shelf micro-controller board (Arduino Mega 2560) combined with 5 custom-made protocol interface boards to pre-process the various signals. The power is provided to the LVPS and the HK boards by two 28 V battery packs; the total power consumption of the electronics (DP and PDM) being 70 W. A detailed description of the DP and its performance is given in (Scotti and Osteria 2016).

### The Trigger

At the time of its design, *EUSO-Balloon* was foreseeing an internal trigger system for UHECR detection on two levels based on the logic developed for the *JEM-EUSO* project (Abdellaoui et al. 2017). The first level trigger is meant to search for a localized signal excess lasting 5 GTUs in a box of 3 × 3 pixels (see 3.2 for more details), while the second one is intended to identify signal excesses lasting 15 GTUs and distributed along linear tracks, short or long depending on the zenith angle of the event. The first level trigger has adaptive thresholds to be calculated every 320 μs in order to keep the rate of spurious triggers due to background fluctuations within a few Hz on the entire PDM. The second level trigger in *JEM-EUSO* has the duty of further reducing the spurious trigger rate at the level of ∼10^−3^ Hz per PDM. In this configuration, both trigger levels are not adapted yet for a balloon perspective. Indeed, the much closer distance of the Extensive Air Showers (EAS) from the detector (20-30 km from the balloon compared to 400 km from the ISS) requires to take into account the ∼10 times faster crossing speed of the signal among pixels.

At the time of the *EUSO-Balloon* flight the *JEM-EUSO* configuration of the second level trigger was successfully implemented on the CCB, however, first level trigger was not implemented in the PDM board due to a lack of resources of the FPGA. For this reason, the second level trigger is bypassed by the acquisition logic; the CPU and the CLKB generating a trigger signal, enabling the acquisition of data. In addition, a trigger synchronized with the 1 Pulse Per Second (1 PPS) signal provided by the GPSR is implemented. This trigger channel is OR-ed with the CPU/CLKB one. The CPU/CLKB and PPS triggers were both used in flight. In the CLK trigger mode, triggers are setup at 20 Hz, synchronous with the GPSR 1-PPS signal, in order to synchronize the acquisition with the light emission from calibrated light sources. These sources are a laser, a LED and Xenon flasher installed on the helicopter that flew under the payload and inside *EUSO-Balloon’s* FoV. Unfortunately, the time synchronization didn’t work as expected during flight, possibly caused by a faulty GPS antenna on the balloon. However, between the CPU/CLKB trigger operating at 20 Hz, and the laser/flasher sequence firing from the helicopter at a rate of 19 Hz, a chance overlap at regular intervals was generated, guaranteeing a number of recorded laser/flasher events (see Sect. 3.1).

After flight the acquired data were processed offline using the first level trigger for UHECR detection that was expected to be implemented on board using the nominal *JEM-EUSO* parameters as well as other variations with shorter number of GTU excess to find a better solution in view of future balloon flights.

### The Optics

**Fig. 4:**
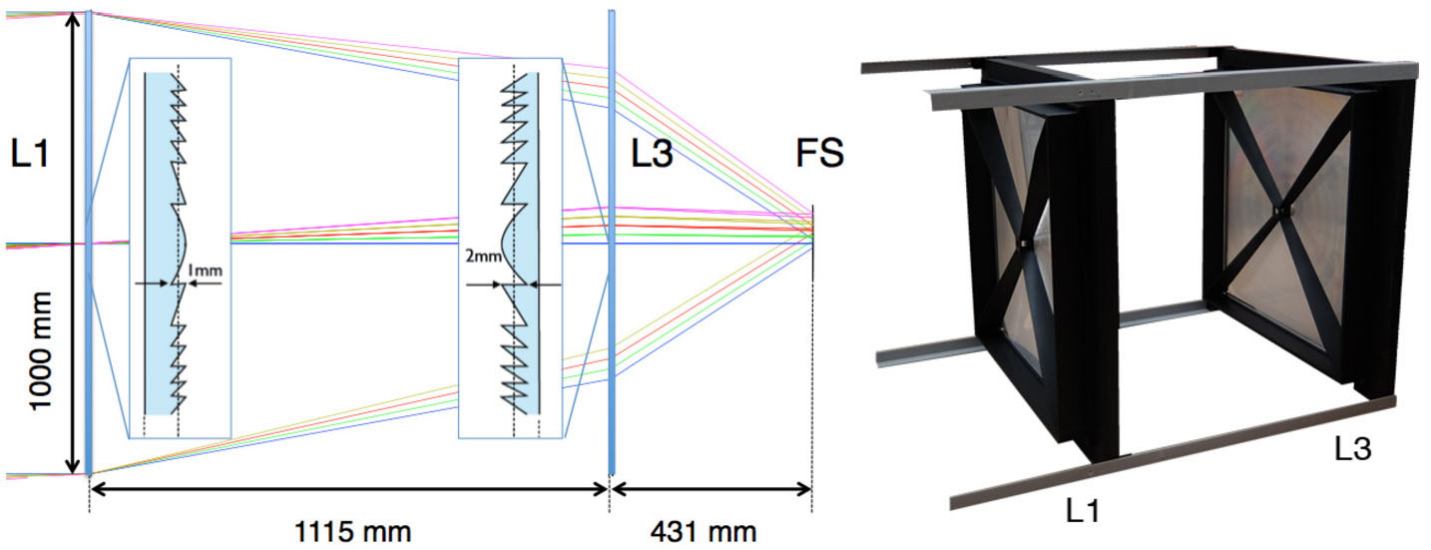
*Left*: ray tracing diagram for the *EUSO-Balloon* optics with the Fresnel Lenses L1 and L3, and the focal surface; the incident rays are at off-axis angles ranging from $0^{\circ }$ (blue) to $1^{\circ }$ (green), 2^∘^ (red) $3^{\circ }$ (yellow) and $4^{\circ }$ (purple); inserts show partial sectional views of L1 and L3; *right*: Optical bench with Fresnel lenses L1 and L3 (8 mm thick PMMA, surface of 1 m × 1 m) mounted onto their fiberglass frames and spiders, and held at a distance of 1.11 m by an optical “sled”

The optical bench containes two square Fresnel lenses made of 8 mm thick PMMA (UV transmitting polymethyl-methacrylate) with a front surface of 100 cm × 100 cm each. The *EUSO-Balloon* optics is designed to resemble the *JEM-EUSO* optics: it is dimensioned to reproduce a UV intensity per pixel comparable to that anticipated for *JEM-EUSO*. A ray tracing diagram and the optical bench are shown in Fig. 4: L1 and L3 are aspherical Fresnel lenses with focal lengths of 258.6 cm and 60.0 cm, respectively (focal lengths are reference values only since single lenses do not produce stigmatic images). The position of L1 can be adjusted along the optical axis within the optical bench. The position of the PDM is adjusted by a translation stage in the instrument booth. Together with the 15 cm × 15 cm focal surface detector the optics provides a FoV of ∼ $11^{\circ }$. A detailed description of the design and manufacturing of EUSO-Balloon optics is given in (Takizawa et al. 2013) and (Hachisu et al. 2013). The measurements of the performance of the fully integrated optical bench (global optical efficiency and point spread function) were performed at IRAP Toulouse and are reported in (Díaz Damian et al. 2019). Compared to *JEM-EUSO* the main difference of this optical system is the absence of the L2 diffractive lens between L1 and L3 to correct for chromatic aberrations.

### The InfraRed Camera

**Fig. 5:**
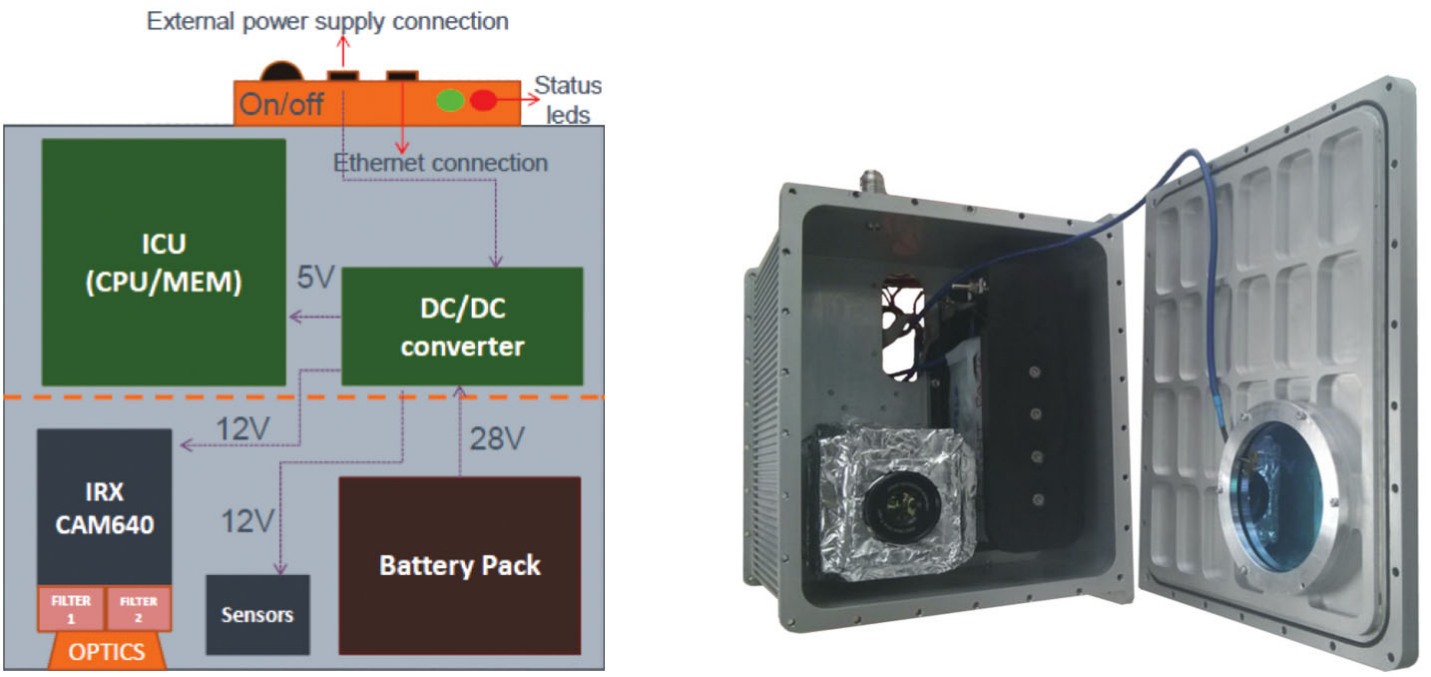
Left: block diagram of the stand-alone IRcam system; right: the rugged watertight aluminium housing (0.4 m × 0.4 m × 0.4 m) of the IRcam with the IRX CAM640 in its lower left side

In order to monitor the cloud coverage and particularly the cloud height, the co-aligned InfraRed camera (IRcam) displayed in Fig. 5 observes the FoV of the main instrument. The general design of the IRcam is similar to the one designed for *JEM-EUSO* (Morales de los Ríos et al. 2013). The camera provides images with a resolution of 640 × 480 pixels, in two wavelength bands centered at 10.8 μm and 12 μm (medium infrared) with 0.85 μm bandwidth, using a ULIS UL 04171 microbolometer and two filters. The field of view of the camera is 45^∘^. The data from the IRcam, along with auxiliary data (temperature, pressure, and humidity), are stored in a RAID1 configuration of two SSDs with 32 GB capacity each. The entire stand-alone IRcam system, including CPU and batteries, is housed in a rugged aluminum box (0.4 m × 0.4 m × 0.4 m) on the outside of the optical bench (see Fig. 1). As the IRcam is not connected via telemetry it relies on recovery of the hard drives after the flight. A detailed description of the IRcam is presented in (Rodríguez Frías et al. 2015), its performance is detailed in (Fernández Soriano et al. 2015).

### The Gondola

The particular configuration of the nadir pointing instrument allowed designing a simple telescope structure serving simultaneously as the balloon gondola. The structure consists of two main modules, the *optical bench* and the *instrument booth* (see Fig. 1), which are both built from 10 mm *Fibrelam* aerospace panels assembled by *Fribrolux* L profiles. *Fibrelam* panels are manufactured from honeycomb that is bonded between composite facing skins; they are light-weight, structurally sound and exceptionally stiff. The dimensions of the fibrelam-telescope itself is 1.21 m × 1.21 m × 2.90 m. The size of the flight-ready gondola is 2.58 m × 2.58 m × 3.7 m, including “crash-rings” and floaters. The overall launch mass of the integrated instrument is 467 kg.

Inside the self-contained, water-tight *instrument booth*, all electronic equipment is mounted on a system of aluminum “shelves” that provides a thermal link to the 1.2 m × 1.2 m aluminum backplate (also called radiator). As the flight chain and harness interface to this backplate, it gets almost all the mechanical stress at the opening shock of the parachutes. The entire gondola is designed to withstand accelerations of up to 15 g along the Z-axis, and 5 g in transverse (X, Y) directions. Besides its role as a structural element, the role of the radiator is to reject the excess heat generated by the electronics to deep space through the top of the instrument booth.

A thermo-mechanical analysis of the instrument was performed to ensure that the various electronic sub-systems, operating in near vacuum conditions, would stay within their admissible temperature range (from −20 ^∘^C to 30 ^∘^C), and to check the mechanical behavior and integrity of the telescope structure in extreme “hot” and “cold” flight scenarios.

The two Fresnel lenses (see Fig. 4) and their mechanical supports are fixed within the *optical bench* so that the optical system is in focus. While L3 is fixed, closing the watertight *instrument booth*, the front lens (L1) can be adjusted along the optical (Z) axis; the PDM is mounted on a translation stage, allowing to adjust the distance between PDM and rear lens (L3).

**Fig. 6:**
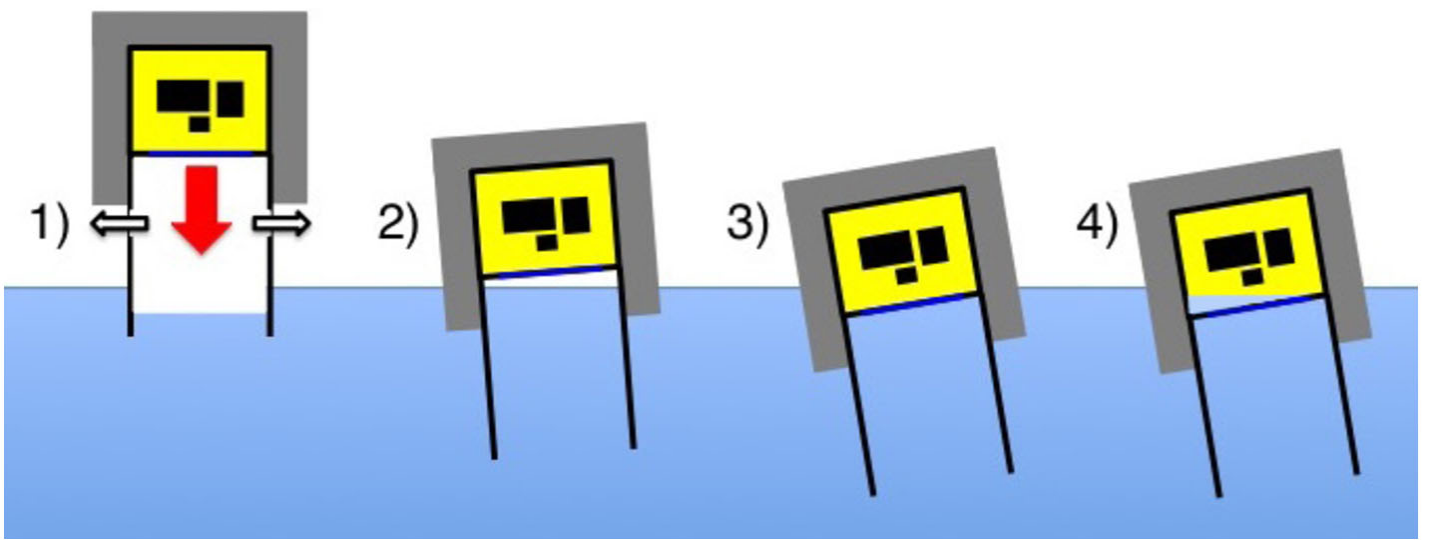
The features making *EUSO-Balloon* survive a water-landing: 1) “deceleration-cylinder”, 2) collar of floaters, 3) watertight instrument booth using the third Fresnel lens (L3) as a porthole, 4) electronics is mounted on a “dry-shelf” above eventual capillary water - see text

Instead of “classic” crash-pads made of layered cardboard-honeycomb, *EUSO-Balloon* uses an ensemble of aluminum “crash-rings”, designed to absorb the kinetic energy on ground impact through inelastic distortion (Fig. 1). *EUSO-Balloon* is deliberately designed to protect all sensitive equipment in the event of a water-landing. A number of independent features (Fig. 6) maximize the chances for recovering the equipment in working conditions: 1) To minimize damage to the payload and to ensure the integrity of the instrument booth at splashdown, efficient deceleration is achieved by using the optical baffle of the instrument as a “deceleration-cylinder” where the pressure of the enclosed air-cushion is passively controlled by calibrated evacuation vents (Fig. 6-1); 2) the entire instrument booth is held above the waterline by a collar of floaters, whatever the orientation of the gondola in the water may be; 3) during splashdown the instrument booth is expected to be at least partially immersed and the submersion might become permanent if the floaters are damaged on impact. Therefore, all electronics is hosted within the instrument booth which is built as a watertight capsule, using the rear lens as a porthole; 4) all sensitive equipment is mounted on a “dry-shelf” with limber-holes, keeping the electronics clear from the inside walls of the instrument booth where capillary water might accumulate.

At the end of its maiden flight in August 2014, *EUSO-Balloon* accidentally splashed down into a tiny solitary lake (barely bigger than two football fields) validating the water-landing capabilities it was designed for in the first place.

## The EUSO-Balloon Flight

**Fig. 7:**
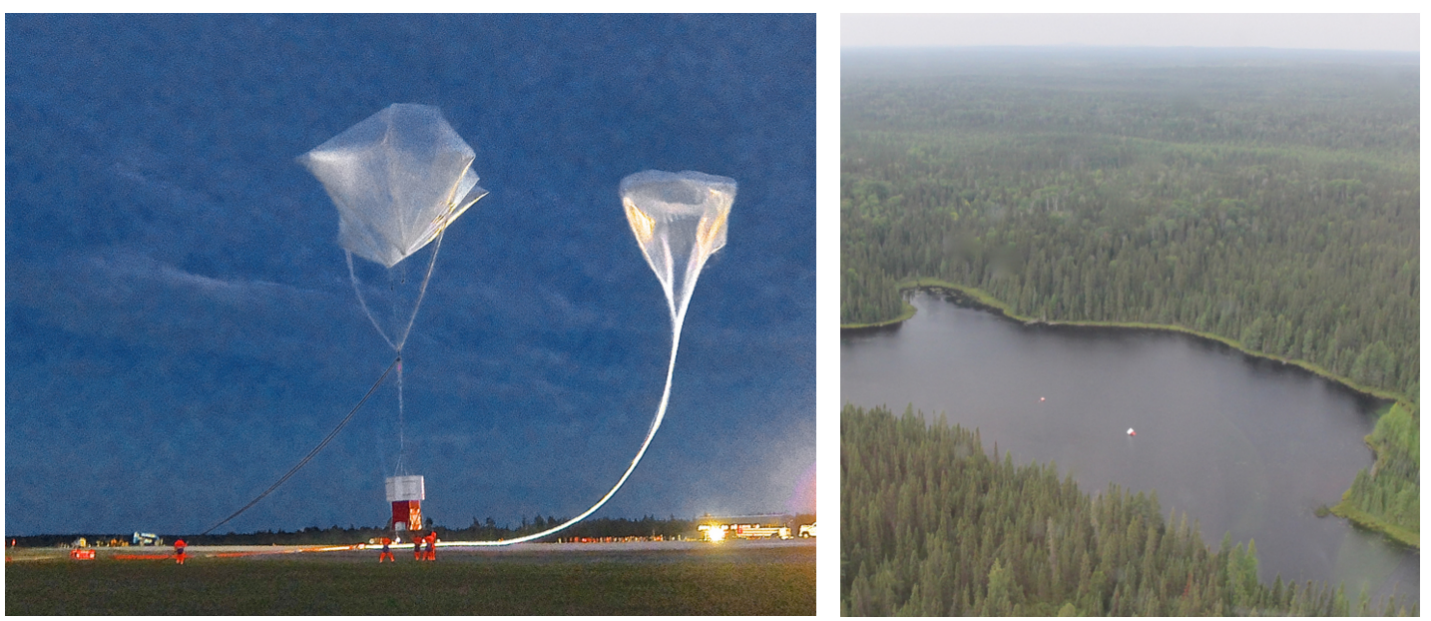
Left: Timmins (Ontario, Canada) Stratospheric Balloon Base, August 25, 2014, 0:53 UTC: the perfect launch of *EUSO-Balloon* by the balloon division of the French Space Agency CNES, the auxiliary balloons (above the payload in the picture) warrant a smooth launch even in case of moderate surface winds. Right: the instrument floating in the middle of “Lake Euso” after splashing down at 8:59 UTC. All systems survived the impact and more than eight hours in the water thanks to a dedicated “water-landing” design

**Fig. 8:**
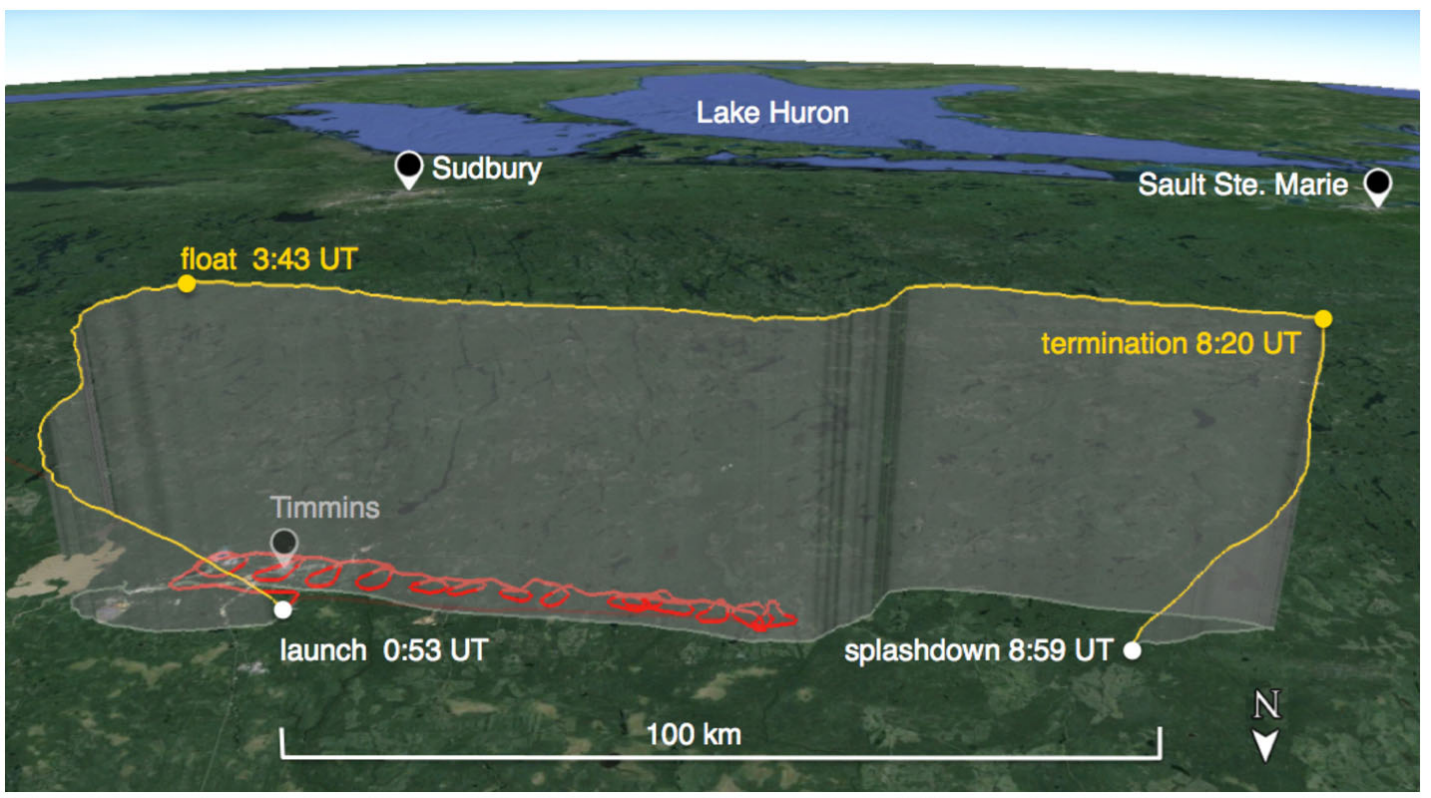
The flight-track of *EUSO-Balloon* on August 25, 2014 (yellow) - float altitude was 38 km. The helicopter carrying the UV laser and two UV flashers followed the balloon for over two hours at an altitude of 3000 m (red)

On August 25, 2014 *EUSO-Balloon* was launched from the Timmins Stratospheric Balloon Base (48.57^∘^ N, 81.38^∘^ W). Thanks to the auxiliary balloon technique routinely used by CNES, the entire launch operation went very smoothly (Fig. 7). The 467 kg payload was lifted from the airfield by a 400,000 m^3^ Zodiac balloon at 00:53 UTC reaching a float altitude of 38.3 km at 03:43 UTC (see Fig. 8 for a map with the flight path). The high voltages of the PDM were switched on at 02:50 UTC at an altitude of 32 km, when the balloon was still on its ascent. Telemetry data rapidly indicated that all systems were operating flawlessly and soon the city-lights of Timmins came into the FoV at the saturation level confirming that the PDM was in good health and was taking UV images. During more than five hours of operation at float altitude, a total of 258,592 data-packets, corresponding to roughly 33 million GTUs, were recorded and written onto the two redundant hard-drives on-board. With the NOSYCA (S-band) telemetry system featuring a data rate of ≤ 1.75 Mbps, about one million GTUs were transmitted to the ground during the flight. The largest part of the science harvest, however, resided on the two on-board hard-drives, and, therefore, relied upon a safe landing and recovery.

**Fig. 9:**
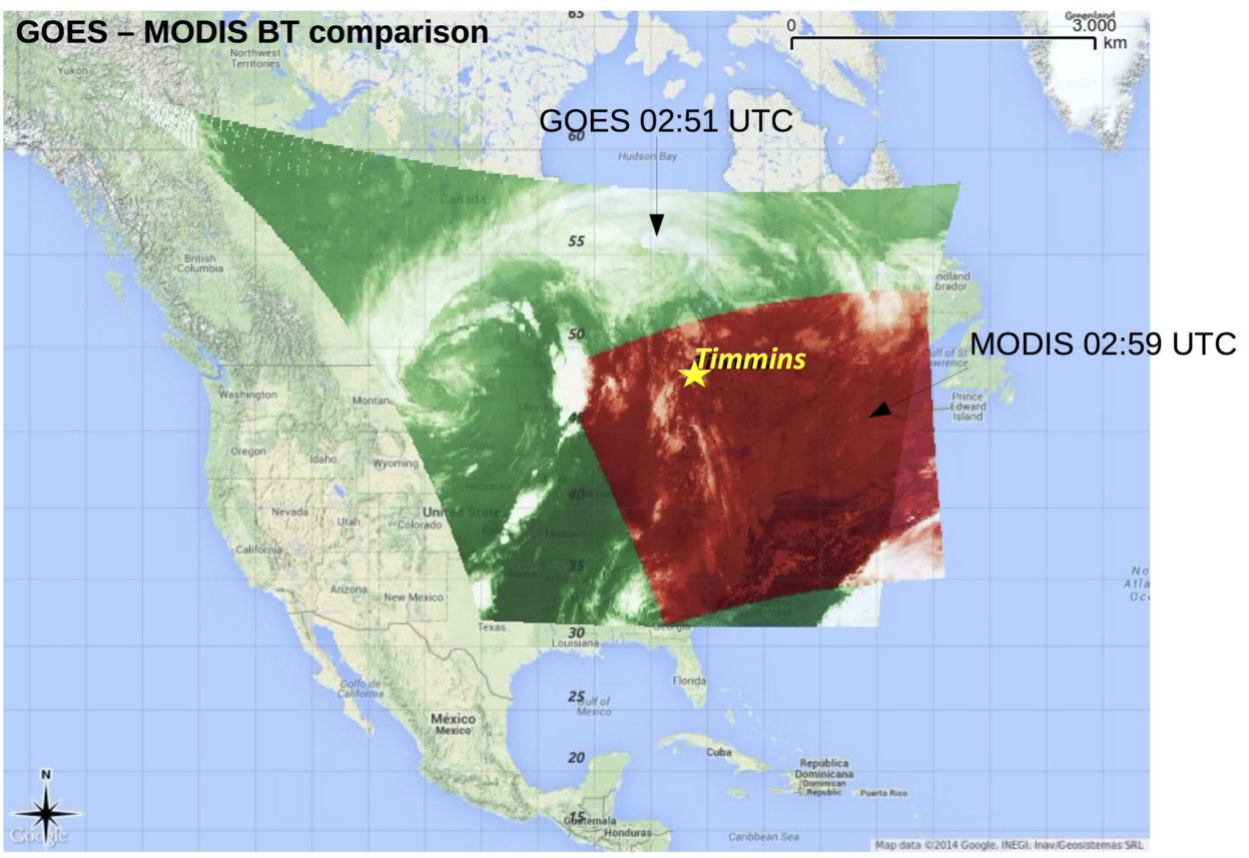
Brightness Temperature (BT) juxtaposition between GOES-13 (green) and MODIS (red) around 3:00 UTC. Note that the size of the image covers a much wider area than EUSO-Balloon trajectory. A star indicates the location of Timmins. It is clear that *EUSO-Balloon* crossed an area of broken clouds during its flight

The moonless night of August 24/25 provided optimal conditions for the study of the UV light intensity: a variety of ground covers were overflown - including different types of soil and vegetation, wetlands, open water, urban and industrial areas. During its flight, EUSO-Balloon crossed areas characterized by scattered and broken clouds at low heights (around 700–800 hPa) and thick ice clouds at higher heights (around 200–300 hPa). This is documented in Fig. 9 which shows a composite image of the data acquired by the NASA MODerate Resolution Imaging Spectroradiometer (MODIS), and by the NOAA Geostationary Operational Environmental Satellite (GOES-13). MODIS (MODIS 2016) is one of the five instruments on board the Terra-satellite flying on a Sun-synchronous polar orbit and provides informations on cloud properties, aerosols and biosphere changes in a wavelength range that goes from 0.4 μm to 14.4 μm; 16 of these bands belong to the IR region. GOES-13 (GOES 2016) hosts a sounder and an imager. The former is a 19-channel radiometer that senses visible, LWIR, medium wave IR and SWIR data to provide vertical atmospheric temperature and moisture profiles, surface and cloud top temperature, and ozone distribution informations. The imager is a five channel radiometer that detects the radiant and solar reflected energy from sampled regions of the Earth.

Towards the end of the astronomical night at 08:20 UTC, the flight was terminated by separating the instrument from the balloon about 100 km west of Timmins. Despite a descent path guaranteeing one of the “driest” landing zones along the flight track, *EUSO-Balloon* and the entire flight train splashed down in a small solitary lake at 08:59 UTC. An adventurous recovery was performed by a crew of trappers and *JEM-EUSO* members with the help of a helicopter. Thanks to its inborn design for water-landings, the entire instrument was undamaged, both lenses were intact, the electronics, IRcam, and the RAID disks had not suffered any water damage and were fully operational.

### The Helicopter Underflight

As it seemed unlikely to detect cosmic ray induced air showers during this short balloon flight, a pulsed UV laser and two types of UV flashers (LED and Xenon) were operated within the FoV of the EUSO-Balloon telescope from a helicopter, which was flying circles along the flight track at an altitude of 3000 m (Fig. 8). The laser produced tracks similar to what is expected from very inclined air showers, while the flashers were meant to provide an absolute calibration source for the instrument. As the flasher light is measured always in the same pixel, this corresponds to EASs developing along the line of sight of a pixel, which means nadir or quasi nadir direction from the balloon perspective. Therefore, the flasher signals were used also to the test the observation of air showers, but in this case with quasi nadir direction, complementing the laser tracks. The Bell 212 helicopter followed the balloon for over two hours. The wavelengths of these sources (355 and 365 nm) were chosen to be close to two of the emission lines of UV fluorescence in air. During this time the sources were fired ∼150,000 times with two energy settings for the laser and four for the flashers (see Adams et al. 2015c; Abdellaoui et al. 2018a for details). The laser energy was changed every two minutes between 15 mJ and 10 mJ and the entire laser and flashers sequence was fired at a rate of 19 Hz. The nominal laser energy corresponds to an EAS energy of about 60 EeV for 15 mJ. This repetition rate was chosen to guarantee random coincidences between the readout of the balloon (20 Hz) and the laser shots. This was necessary because the synchronization system (see Sect. 2) did not work as expected.

The light sequence (flashers and laser) was set up in the following way. First, a UV LED was fired for 30 μs (12 GTUs) with increasing luminosity to achieve a projected number of photoelectrons at the PDM level raising from ∼1 to ∼50 counts. The sequence of LED intensities was kept constant during the entire flight. This light signal appeared on the focal surface as a static source and could be used to determine the position of the helicopter in the FoV. About 62.5 μs (25 GTUs) after the end of the LED signal, a laser shot lasting 7 ns was fired. The laser event took at maximum 25 μs (10 GTUs) to cross the entire FoV of the telescope. A Xenon flasher was finally discharged ∼5 μs after the laser shot for an 20 μs (8 GTUs) duration. The variable light intensity of the Xenon flashers reached a maximum after the first 7.5 μs (3 GTUs) and then decreased for the remaining time. Four different absolute intensities were used to mimic different EAS energies.

**Fig. 10:**
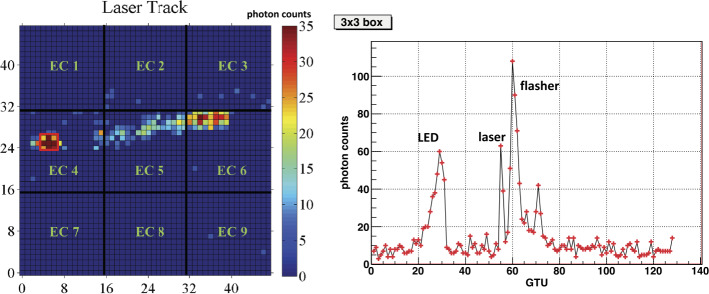
Left side: Image of one helicopter event obtained by integrating the counts in each pixel for the whole packet=1960 or run=043202 (128 GTUs). This event includes all three components: UV-LED and Xenon-flasher signals as well as laser track which extends up to coordinates (X=39; Y=31). A threshold is applied to the minimum signal level to emphasize the location of the track. The UV-LED and Xenon-flasher signals are centered around a pixel: axis of abscissae X=5; axis of ordinates Y=25. Right side: The number of photon counts recorded in the 3×3 pixel-box centered around (X=5; Y=25) during the entire packet. Figure adapted from (Suino et al. 2015)

An example of such a light sequence is displayed in Fig. 10. The integrated number of counts in a typical packet (run=043202, packet=1960) in which all the light sequence was imaged is shown in the left plot. The LED and Xenon flasher signals are located around a pixel with coordinates (X=5; Y=25). The laser tracks extends up to coordinates (X=39; Y=31). Due to the lower sensitivity of a few MAPMTs only a portion of the track is clearly visible with the applied thresholds. The evolution of the signal in the 3×3 pixel-box centered around (X=5; Y=25) during the entire packet is displayed in the right plot. The LED signal appears between GTUs 19–31, followed by the laser shot at GTUs 55–56 and by the Xenon flasher between GTUs 58–65. An afterpulse from the Xenon flasher occurs between GTUs 70–73. Taking into account also this afterpulse the entire sequence lasts ∼140 μs.

### In-Flight Instrument Performance

The main objective of the flight was to operate a full scale end-to-end test of most of the key technologies and instrumentation which are expected to be employed in *JEM-EUSO*. Different configurations of the MAPMT gains and thresholds for the photon-counting were applied. The acquisition system performed rather well with an integrated data taking period of 15,300 s out of about 18,900 s at float altitude (∼81% of the total acquisition time at float) (Scotti and Osteria 2016). The trigger was provided at a rate of ∼20 Hz. 258,592 events were recorded. Each event had an individual integration time of ∼320 μs (128 GTUs), and with a forced trigger rate of 20 Hz, the instrument only recorded data for 6.4 ms out of every second. As a consequence, the total integrated time was 83 s across the entire flight taking into account also the acquisition time devoted to “health-checks” of the instrumentation as well as the off time to restart the acquisition procedure between runs.

The acquisition time was uniformly distributed across the 5 hours of the flight and allowed for a measurement of the light intensity in various locations.

The front-end electronics and the MAPMTs behaved rather well. Only 1 out of 9 ECs had a failure (EC1 in Fig. 10). In total only 5 out of 36 MAPMTs could not be used for data analysis. A detailed calibration of the 36 MAPMTs for a total of 2304 pixels was performed before the flight and repeated after recovering the instrument. Details are reported in (Moretto et al. 2015). Among the active pixels, 650 were chosen, based on their performance, to get a measurement of the UV light intensity. For these pixels the absolute uncertainty on their efficiency was < 5%, leading to a relative uncertainty of 7% for the pixel efficiency at 378 nm. The average efficiency of these pixels measured at 378 nm was (19.3 ± 0.1)%. This value includes the detection efficiency of the MAPMT and the transmission of the BG3 filter. The efficiencies of the pixels at other wavelengths were relatively rescaled based on the typical quantum efficiency curve of this MAPMT and of the BG3 transmission curve as a function of the wavelength. A detailed model of the electronics response was developed in order to have a precise estimation of the detector sensitivity and the photon-counting rate, after subtracting the electronic noise of the system (Dagoret-Campagne et al. 2015).

The performance of the optics system was carefully studied, being one of the key parameters in a refractive telescope such as *JEM-EUSO* (Díaz Damian et al. 2019). Indeed, the optics throughput and its point spread function can vary significantly as a function of the wavelength, therefore, it was necessary to verify its performance experimentally. For this reason, it was characterized at wavelengths of 313, 334, 365 and 405 nm and incidence angles of 0.1^∘^, 2.3^∘^, 3.3^∘^ and 4.5^∘^ by using a UV light source placed at a hyperfocal distance from the optics. The measured efficiency of the optics varied between 6% and 34% depending on wavelength and incidence angle. The estimated efficiency for the observation of the main EAS fluorescence emission lines varied between 21% and 28% depending on the incidence angle.

The pre-flight work allowed to optimize the optics configuration for the balloon flight, i.e. the placement of the optical elements and PDM that gives the optimal Point Spread Function (PSF) and the Photon Collection Efficiency (PCE). However, the campaign was not exhaustive due to the tight deadlines imposed by the balloon launch date. The post-flight work allowed to measure more exhaustively the optics performance. The goal of the post-flight characterization was to understand the PCE of the optics flight configuration as a function of angle. Moreover, in a subsequent study where a different experimental setup was used to explicitly study the diffraction patterns (details can be found in (Díaz Damian et al. 2019)) it was determined that the lenses diffuse light by diffraction due to residual fabrication features in the lenses surface, especially in the middle and high spatial frequency regimes. The characterisation results could be understood by combining a simple ray tracing code for refraction/reflection with a semi-empirical term for diffusion obtained experimentally.

Taking into account both the optics and focal surface efficiencies, the global efficiency of the instrument turned out to be of the order of (4–5)% for point-like sources, depending on wavelength and incidence angle for the characterized wavelengths around the main EAS fluorescence emission lines. The characterisation of the EUSO-Balloon optics allowed to find the best lens positions in order to define an optimal telescope configuration prior to the balloon flight. This contributed to the fulfilment of the primary objectives of the mission.

**Fig. 11:**
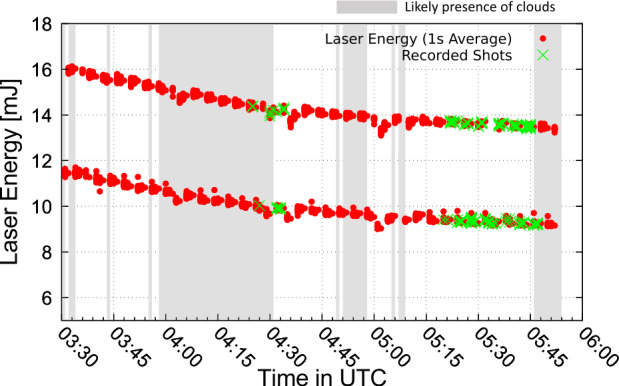
Red dots: energy of all fired laser shots averaged over 19 shots (1 s). Green crosses: shots recorded by *EUSO-Balloon*. Grey regions indicate the likely presence of clouds. The laser energy was decreasing due to heating of the laser itself during the helicopter flight. Figure adapted from (Abdellaoui et al. 2018a)

The data collected by *EUSO-Balloon* were used to fine tune offline the *JEM-EUSO* first level trigger algorithm which was then employed on board subsequent missions (Abdellaoui et al. 2017). The employed trigger algorithm requires a locally persistent signal above the average background lasting a few GTUs. In this trigger level, pixels are grouped in cells of $3\times 3$ pixels. A cell issues a trigger if it satisfies the following conditions: a) for a certain number of GTUs ($N_{\mathrm{ctd}}$) in a group of consecutive GTUs ($N_{\mathrm{pst}}$), there is at least one pixel in the cell with a number of counts equal to, or higher than, a preset threshold, $n_{\mathrm{thr}}^{\mathrm{pix}}$; and b) the total number of counts integrated in the cell is higher than a preset value $n_{\mathrm{thr}}^{\mathrm{cell}}$. $N_{\mathrm{ctd}}$ and $N_{\mathrm{pst}}$ are set to 3 and 5 GTUs, respectively, while $n_{\mathrm{thr}}^{\mathrm{pix}}$ and $n_{\mathrm{thr}}^{\mathrm{cell}}$ are set as a function of the average background level to keep the rate of spurious triggers around 1 Hz per EC. Based on this algorithm, 275 events in the two time windows between 04:17 - 04:34 and 05:16 - 05:45 (see Fig. 11) were classified as “helicopter events” (Suino et al. 2015) as they passed the trigger criteria in at least two independent EC units in a time difference compatible with the crossing time of the laser track through the FoV of the telescope. This resulting number of events is within expectations, taking into account a chance overlap at regular intervals between the 20 Hz readout of the balloon and the laser as well as some periods of cloud obscuration. Details of this estimation are reported in (Abdellaoui et al. 2018a).

## Summary of Science Results

This section describes the main scientific results of *EUSO-Balloon* flight which were the characterization of the UV light intensity in different locations and atmospheric conditions, the imaging and reconstruction of the artificial lights which were supposed to mimic EAS tracks, and the unexpected signals which were detected along the flight.

In order to provide quantitative results and comparisons with expectations, the configuration of the *EUSO-Balloon* instrument was incorporated in the two different packages which are officially adopted by the *JEM-EUSO* collaboration as simulation and analysis frameworks: the EUSO Simulation and Analysis Framework (ESAF) (Mernik et al. 2013) and the $\overline{\text{Off}}\underline{\text{Line}}$ package (Panico et al. 2015). Both ESAF and $\overline{\text{Off}}\underline{\text{Line}}$ were expressly developed for experiments devoted to the observation of UHECRs. While $\overline{\text{Off}}\underline{\text{Line}}$ was originally established for the Pierre Auger Observatory and only subsequently adapted to the *JEM-EUSO* case, ESAF was developed for a *JEM-EUSO*-like space-based observatory. The main goal of both softwares is to perform the simulation of the UHECR event, its detection and the relative EAS parameter reconstruction, or simply the reconstruction of the air shower parameters in case of events detected by the instrumentation. As an example of their utility applied to *EUSO-Balloon*, the dimmest events of the flashers were used to estimate the minimum EAS energy that would have produced such a light intensity, which turned out to be E ≳5×10^18^ eV for EASs developing in nadir direction, adopting the *EUSO-Balloon* configuration implemented in ESAF. Moreover, the data collected by *EUSO-Balloon* were carefully studied (Bacholle et al. 2015b) in order to estimate the capability for detecting EASs with a much longer flight, such as the EUSO-SPB1 mission (Wiencke and Olinto 2017) still using ESAF simulations. Their applications for the science results presented in this section are described in the following.

### Estimation of UV Background Light

The main scientific objective of the *EUSO-Balloon* flight was the measurement of the background UV light intensity. This is relevant for *JEM-EUSO* as it is one of the key parameters for estimating the exposure curve as a function of energy (Adams et al. 2013). Balloon measurements have been performed in the past by BaBy (Giarrusso et al. 2003), NIGHTGLOW (Barbier et al. 2005) and a JAXA payload (Sakaki et al. 2007). However, *EUSO-Balloon* uses a very different approach. It is based on an optical refractive system with very fine spatial and temporal resolutions; the filter has a large bandwidth (∼290 - 500 nm) which requires a careful computation of the optics and detector response to translate the detected counts into an absolute measurement. From the point of view of the capability of a space-based observatory for UHECRs, the essential point is the number of background counts per GTU at the pixel level. This is the pedestal that should be dark enough to permit detection of an UHECR track. A detailed description of the analysis and results to infer the background UV light intensity observed by *EUSO-Balloon* is reported in (Abdellaoui et al. 2019). Here we summarize the key aspects and results of the analysis.

**Fig. 12:**
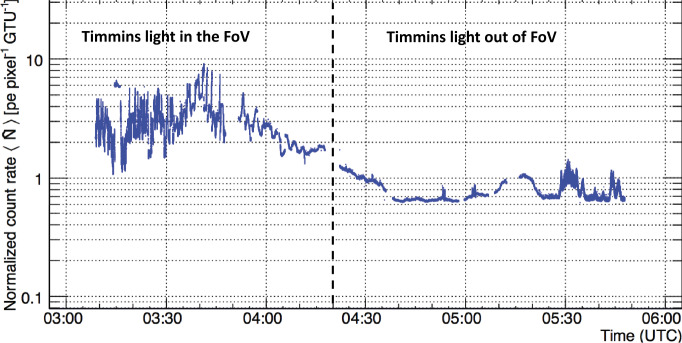
Average normalized count rates ${\langle \hat{N}\rangle }$ as a function of the packet time. Several breaks are present due to the interruption of the measurements necessary to switch between different data acquisition modes. Figure adapted from (Abdellaoui et al. 2019)

Figure 12 shows the average normalized count rates ${\langle \hat{N}\rangle }$ as a function of the packet time. Several breaks are present due to the interruption of measurements necessary to switch between different data acquisition modes which were part of the technological tests foreseen for this flight. The conversion of the digital counts in each pixel into UV intensity has to take into account many aspects. Among them, the most relevant are: entrance aperture of the optics and its throughput, MAPMT detection and filter efficiencies, the pixel’s FoV and GTU duration. Many of these parameters are wavelength dependent. An accurate determination of all these parameters was performed and is discussed in (Dagoret-Campagne et al. 2015; Moretto et al. 2015; Díaz Damian et al. 2019).

In *EUSO-Balloon*, only the back-scattered light from the airglow and extraterrestrial light contributes to the measured diffuse light. The reflectivity of the clouds is expected to be higher than clear atmospheric conditions. Thus, the time interval and area with lowest count rates is assumed to represent clear atmosphere. Such conditions were assumed to be present between 04:38 and 04:52. Based on the average of the distribution in that time window, the reference ${\hat{N}_{0}}$ value is ∼0.65 counts pixel^−1^ GTU^−1^. Between 04:20 and the end of measurement, when the artificial lights of Timmins and surroundings were out of the FoV, the count rate varied within a factor of ∼2. This gives the maximal difference of UV intensity between clear and cloudy atmospheric conditions during the flight.

Ray trace simulations were performed using the $\overline{\text{Off}}\underline{\text{Line}}$ code to translate ${\hat{N}_{0}}$ in absolute intensity ($I_{0}$) values. In the area with no artificial light sources, based on the airglow (Dekker et al. 2000; ESO-UVES 2016) and starlight (Leinert et al. 1998) models, the measured count rate from the diffuse light under clear atmosphere conditions corresponds to $I_{0} \sim 300~\text{photons}$ m^−2^ sr^−1^ ns^−1^ in the 300–500 nm band. The value obtained by *EUSO-Balloon* is comparable to the values obtained by previous experiments. This shows that the behavior of the instrument is understood in a reasonable way, despite the complexity of such a measurement by an experiment with a very large bandwidth of sensitivity. Note, anyway, that airglow is a dynamical phenomenon, therefore, its intensity varies in time and position of the Earth as well as by the influence of geomagnetic activity and atmospheric tides (Pfaff 2012). Moreover, the average count per pixel is in the expected range for *JEM-EUSO*, therefore the focal surface response was tested at a level of illumination foreseen for a space mission, which was one of the main goals of the *EUSO-Balloon* mission.

**Fig. 13:**
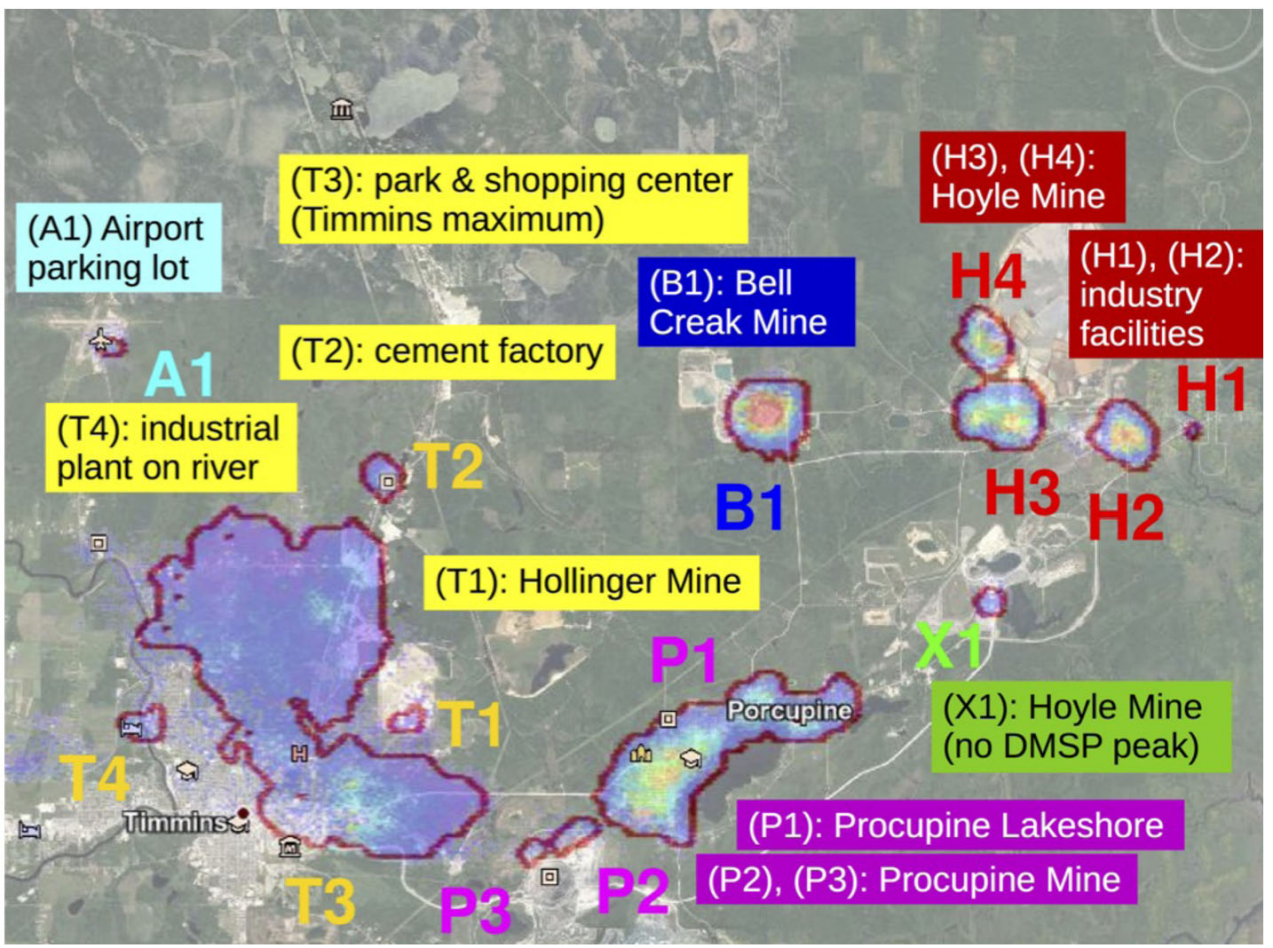
The contoured areas are those detected by EUSO-Balloon with significant light intensities. They are superimposed with a satellite image (Google Maps 2016) of the Timmins area. A good match between the two images is evident

Another important capability is to match the detected artificial lights with those expected from satellite images. This is useful in estimating the exposure of a space-based experiment because it allows autonomous recognition of the areas to be excluded from the calculation. Figure 13 displays one such example showing the locations with particularly high light intensity. They match with ground-based sources according to satellite images (Google Maps 2016).

### UV Intensity in Different Atmospheric Conditions

**Fig. 14:**
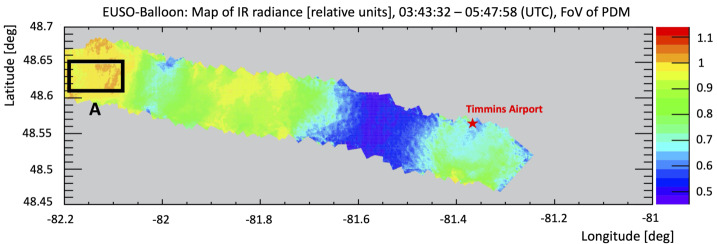
Geographical map of the IR radiance along the flight path (i.e. from “right to left”, as the balloon was carried towards the west by the winds in the stratosphere). The map is created by averaged values for particular positions. The values were changing in time due to movement of clouds and motion of *EUSO-Balloon*. The displayed values are relative to the mean value of IR radiance over reference area “A”. Figure adapted from (Mackovjak et al. 2015)

In the previous section, the UV intensity and imaging of *EUSO-Balloon* is quantified focusing on the clear atmospheric conditions. These conditions were mainly determined by the reports of the helicopter’s pilot flying below the detector. However, as mentioned earlier, the *EUSO-Balloon* instrument flew a bi-spectral IR camera operated as a stand-alone device during the flight to obtain the Cloud Top Height (CTH) and cloud coverage in the FoV by using two LWIR bands centered at 10.8 μm and at 12 μm (Rodríguez Frías et al. 2015). During the flight duration, about 350 images were recorded, one every 80 s. In this section, the main result of a comparison between the UV intensity and IR radiance, both in relative units, is summarized. The detailed analysis is reported in (Mackovjak et al. 2015). UV and IR values were normalized to the average ones recorded in the area named “A” in Fig. 14. This area partly corresponds to the region where the helicopter’s pilot indicated clear atmospheric conditions for most of the time.

**Fig. 15:**
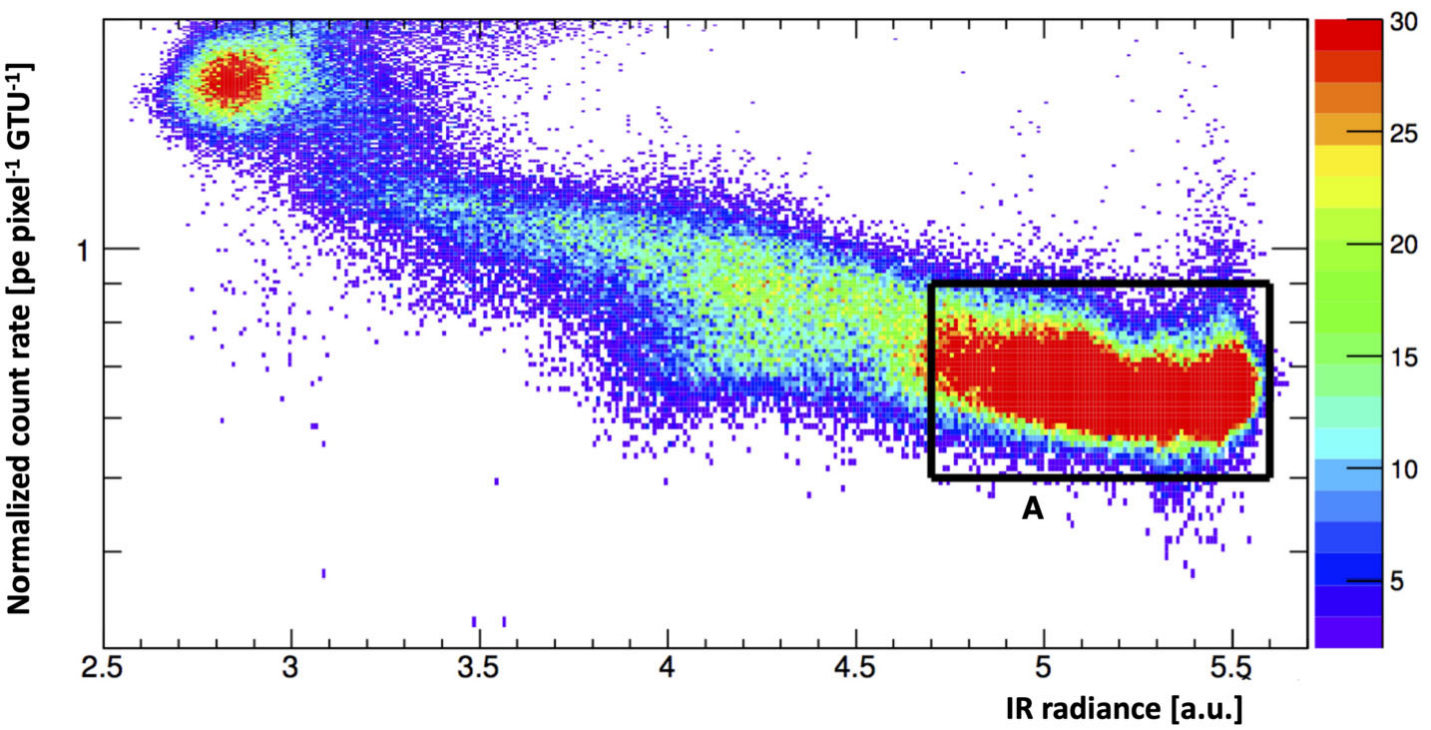
The cross-correlation of IR radiances and UV intensities of all pixels from the UV and IR maps. The color scale represents the number of overlapping pixels in the scatter plot which is arbitrarily truncated at 30 units. The selected rectangle indicates conditions that are suitable for the detection of EASs - cloudless atmosphere without man-made lights and corresponds to area “A” already defined in Fig. 14. The logarithmic scale is used to improve the visualization of the anti-correlation presented in the text. Figure adapted from (Mackovjak et al. 2015)

First of all the IR camera shows high radiance in the second part of the night, which means clear atmosphere, while clouds are present on the outskirts of Timmins (first part of the night) in general agreement with the pilot’s reports. Figure 15 shows the cross-correlation of IR radiances and UV intensities of all grid points from UV and IR maps. The color scale is used only to give an idea of the number of overlapping pixels in each point of the scatter. This number is arbitrarily truncated at 30 units. The points in the scatter plot with UV intensities higher than 2 counts pixel^−1^ GTU^−1^ have no physical meaning as they are produced by man-made light sources (see Fig. 12).

In general, there is an anti-correlation between the UV flux from a given direction and the IR radiance from the same direction in presence of clouds. In such a condition the UV intensity can rise up to a factor of about two, while this effect is not present in case of clear atmospheric conditions. A qualitative explanation for the anti-correlation is that clouds with larger optical depth are more efficient in scattering the UV radiation and producing an albedo which increases the overall intensity of the UV background in cloudy conditions. UV radiation is absorbed in the atmosphere and higher altitude clouds have a higher albedo (at equal optical depth). Higher clouds are also colder and produce lower IR radiance. In general, a combination of the measurement of IR emission and UV albedo of the clouds provides a tool for characterization of the clouds. This should improve the quality of reconstruction of EASs occurring in cloudy atmospheric conditions, and allow “masking out” the regions in which the quality of UHECR data would not be acceptable.

Space-based measurements benefit from this UV-IR anti-correlation since IR cameras coupled with the main UV sensor can estimate the cloud altitude and optical depth, which aids the reconstruction of UHECR events. As an example, the height of the shower maximum in atmosphere strongly depends on the zenith angle of the EAS. Therefore, by having some indications of the range of cloud-top heights in the FoV, it would be possible to apply quality cuts which exclude the reconstruction of the EAS energy for all those EASs whose inclinations are such that the shower maximum is expected to be below the cloud-top height. Alternatively, it would be possible to assign a lower limit to the shower energy, if part of the light intensity is absorbed by the cloud. In some analyses related to the correlation with astrophysical sources, it is important to set a lower limit on the energy of the events, as these correlations exists only above certain energies (typically above 3–5×10^19^ eV) (Aab et al. 2018). Therefore, a good reconstruction of the EAS direction and a lower limit on the energy estimation would be already beneficial. On the other hand, the knowledge of a potential presence of clouds, could become an exclusion cut for the reconstruction of the UHECR energy spectrum where it is important to have a good measurement of the EAS energy and of the instantaneous exposure of the detector.

### Cloud Top Height Retrieval

Reliable information on cloud properties, such as the CTH, is crucial to properly evaluate the exposure of a space-based detector and to reconstruct air showers. In *JEM-EUSO* different methodologies were developed to retrieve the CTH from IR images (Merino et al. 2015; Anzalone et al. 2019). One technique is based on stereo-vision algorithms and requires two different views of the same scene. The height reconstruction relies on accurate image analysis, depends on the geometry of the system, and does not need extra atmospheric information. Another technique, which is presented in detail below, converts Brightness Temperatures (BTs) into CTHs by using vertical temperature profiles obtained from the Weather Research and Forecasting (WRF) model (Skamarock et al. 2008), a mesoscale numerical weather prediction (NWP) model that, in absence of available radiosounding data, allows the reproduction of real-time atmospheric profiles through post-event simulations performed at any time.

The WRF model was applied to the *EUSO-Balloon* observations to check its reliability in evaluating atmospheric vertical profiles to determine the height of clouds eventually detected by the IR camera. At first, the accuracy of the WRF simulated profiles were tested by comparing them to radiosounding data observed close to the balloon flight path. Then, the analysis was extended to the entire *EUSO-Balloon* scene by testing the simulated profiles on the satellite spectroradiometer MODIS, used as a reference sensor. Finally, by applying a methodology based on WRF, the brightness temperature information retrieved by IR camera images was translated into CTH (an example is reported in the following section). The details of these analyses can be found in (Merino et al. 2015) and (Tabone et al. 2015).

Three radiosounding stations close to the *EUSO-Balloon* flight were considered: Moosonee, Green Bay and Gaylord. The soundings were recorded on August 25, 2014 at 00:00 UTC. A fourth real vertical temperature profile, recorded in the first 40 minutes of flight by a thermometer installed on the balloon, until it reached about 17 km of altitude, was used in addition. Here, the WRF model version 3.6 was used to simulate the atmospheric conditions over the entire balloon flight. The model was initialized by global analyses provided by the ECMWF (European Centre for Medium-range Weather Forecasts) global model (ECMWF 2016) with grid-spacing of $0.125^{\circ } \times 0.125^{\circ }$. The unique MODIS data available for the entire balloon flight were the ones observed in the scan that occurred above Canadian lands on August 25, 2014 at 02:59 UTC. The MODIS Cloud Top Pressure (CTP) and the MODIS Cloud Top Temperature (CTT) products were taken into account.

Atmospheric vertical temperature profiles provided by the radiosoundings recorded close to (and in) the *EUSO-Balloon* path flight during the experiment were compared with the WRF model profiles simulated at the same time and in the same locations. To quantitatively evaluate the temperature deviations between real and simulated profiles, the RMSE (Root Mean Square Error) values for each comparison were computed. All the errors turned out to be within $1^{\circ }C$ of temperature deviation, suggesting that WRF simulated profiles are in good agreement with real observed data.

The accuracy in using modelled atmospheric vertical profiles to evaluate the CTH (or CTP) from the CTT was then assessed on the *EUSO-Balloon* scene observed in (near) real-time by the satellite sensor MODIS. MODIS provides both CTP and CTT, thus the comparison between the MODIS estimate of CTP, considered as very accurate, to the CTP obtained by applying the WRF-simulated temperature profiles to the CTT. This helps to evaluate the goodness of the CTP (or CTH) retrieval procedure. The potential for using a NWP model is that for each surface grid-point a specific atmospheric vertical temperature profile can be retrieved. Moreover, using a mesoscale model such as WRF, allows the use of high resolution grid boxes (i.e. 3 km resolution), which produce the best profiles. To each single element of the CTT matrix its closest WRF simulated vertical profile can be applied and the CTP of the entire image is thus evaluated. Also, the performance in CTH retrieval of the other three temperature profiles was investigated: the profiles were taken from the Moosonee and *EUSO-Balloon* soundings and from a Standard Atmosphere model developed for mid-latitudes. In this case the same real or standard profile was applied to the whole CTT matrix.

**Fig. 16:**
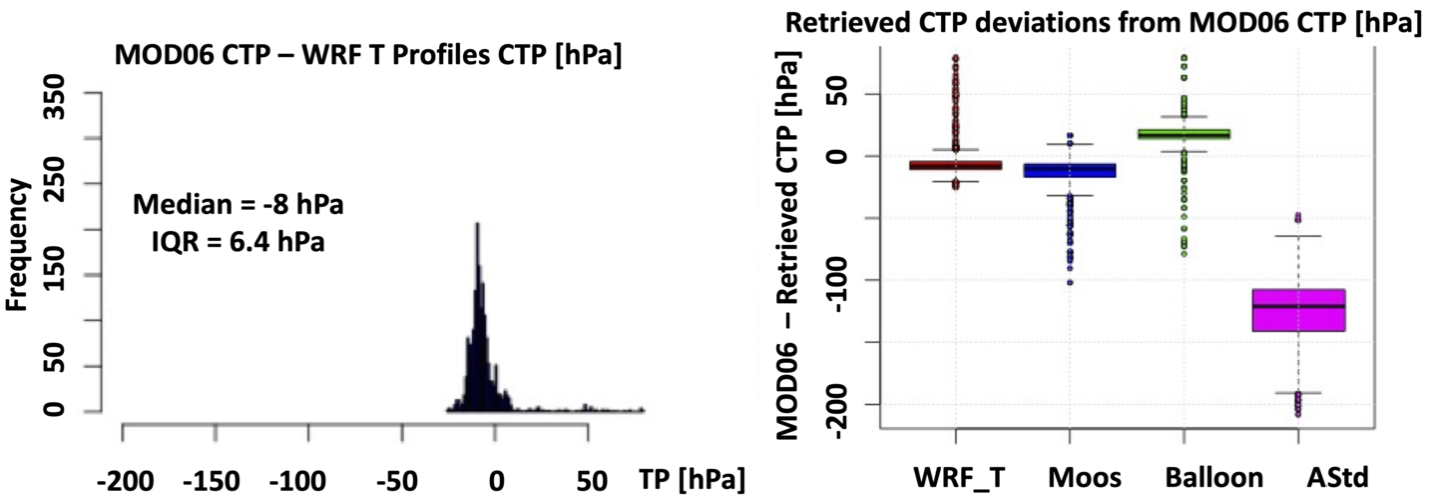
Left: Histogram of the discrepancies from MODIS CTP and the CTPs retrieved using WRF profiles. Right: Boxplot of the discrepancies from MODIS CTP and the CTPs retrieved applying the WRF (WRF T), Moosonee (Moos), EUSO-Balloon (Balloon), and US Standard Atmosphere (Astd) profiles to the MODIS CTT image. Figure adapted from (Tabone et al. 2015)

Figure 16 (left side) shows the histogram of the discrepancies from MODIS CTP and the CTPs retrieved using WRF profiles, while Fig. 16 (right side) shows the boxplot of the difference between the MODIS CTP and the CTPs retrieved using the profiles aforementioned. From the InterQuartile Range (IQR) value and the median, it is clear that the best performance in pressure (height) retrieval is achieved using the WRF simulated profiles applied element-by-element to the CTT matrix. By far the worst performance is achieved by using a standard atmospheric profile, it is clear how the usage of the WRF simulated ones can improve the CTP (CTH) retrieval.

**Fig. 17:**
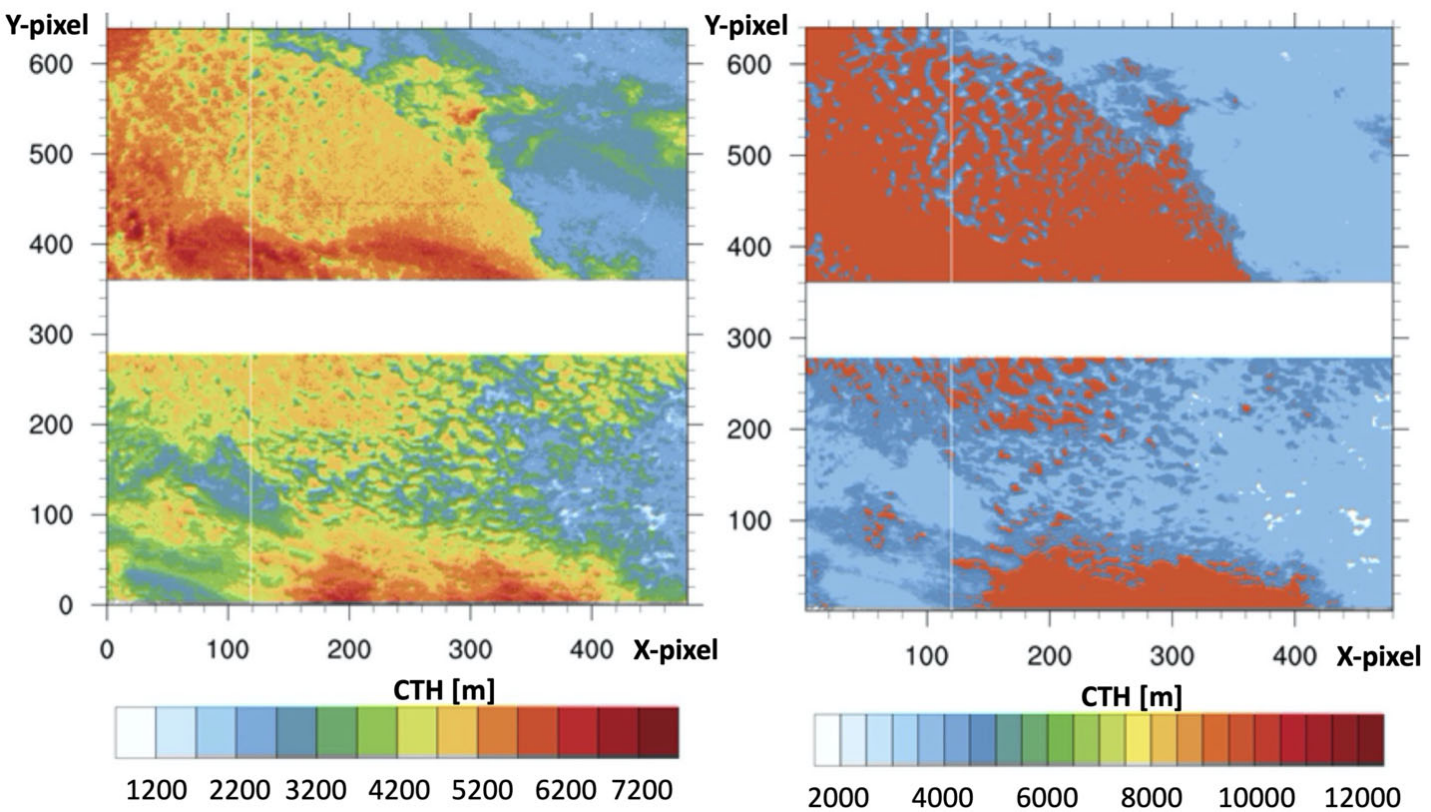
CTH (expressed in meters) of *EUSO-Balloon* scene for 07:39 UTC (legend at the bottom). Left: CTH of algorithm assuming clouds as black bodies. Right: CTH final, including WRF corrections. X- and Y-axis represent the pixel number. The FoV at ground is order of 23 × 32 km^2^. Figure adapted from Merino et al. (2015)

In (Merino et al. 2015) the same approach was applied to one scene of the IR camera data of *EUSO-Balloon*. In this case, the brightness temperature data obtained by the IR camera were translated directly into CTH using WRF vertical profiles without the intermediate step of determining the CTT. Figure 17 shows the CTH of the *EUSO-Balloon* scene taken at 07:39 UTC by the IR camera. On the left side, clouds are considered as black bodies and CTHs vary between 4–7 km. When the WRF corrections are applied, the CTH raises up to 10 km (right side). This shows the importance of using NWP models for this type of study.

### Event Reconstruction and Imaging Performance

The events recorded by *EUSO-Balloon* originating from sources on board the helicopter were revealed to be useful to understand the system’s performance (optics, photo-detector, and front-end electronics), and to test the capability of detecting and reconstructing EAS-like events. The *EUSO-Balloon* configuration was implemented inside the *JEM-EUSO* $\overline{\text{Off}}\underline{\text{Line}}$ package (Panico et al. 2015). Laser tracks were used to test the reconstruction algorithms. A detailed description of this analysis, including the methodology, is reported in (Abdellaoui et al. 2018a).

#### Track Identification

**Fig. 18:**
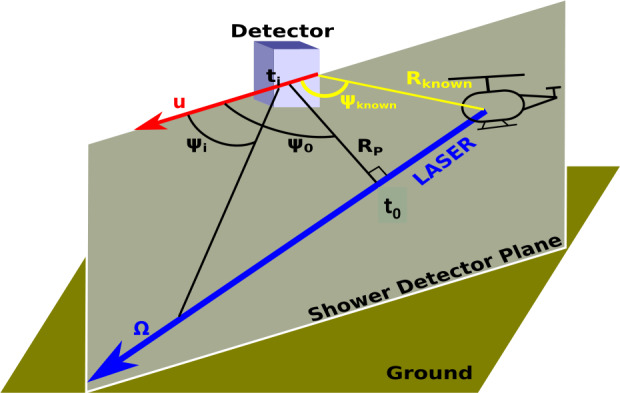
Illustration of the reconstruction of the geometrical direction of the laser tracks fired from the helicopter using the observables from the balloon. The two parameters $t_{0}$ and $\Psi _{0}$ represent the time at which the laser track reaches the closest distance to the detector, and the angle between the detector’s direction of movement and the line of closest distance between the track and the detector, respectively. If the position of the light source is known, these are the only two parameters that need to be determined. Figure adapted from (Abdellaoui et al. 2018a)

A track is a set of neighbouring pixels following a line which are activated during a few consecutive GTUs. To identify tracks, a two-level identification-algorithm was implemented in the offline data analysis. The first level is a simple threshold identification. The average background per pixel over 128 GTUs is calculated. If at least 25 pixels have a value 5 sigmas above this average background the event is selected for further analysis. The event is then processed by the second level identification-algorithm. As a first step a cluster of pixels is created. A pixel is added to a cluster if its signal is 5 sigmas above its background and it is not further away than three pixels from the cluster. Next, the algorithm performs a linear time fit on the clusters to identify tracks in the data. First of all a shower detector plane is defined (see Fig. 18), then a trial nominal direction is estimated. In the next step the expected time for the signal to reach the detector is calculated for each pixel based on the region of the event axis to which it points. The difference between the expected and the observed time is compared and the parameters are adjusted to minimize time differences across the camera using the $\chi ^{2}$ minimization method. The geometry with the minimum difference is used to reconstruct the shower axis. With this algorithm, 205 tracks were found.

For the reconstruction analysis, a track length of 4 GTUs was required to ensure no false positives. With this constraint, 190 events were found. This number is lower than what was found by the trigger logic run offline (see Sect. 3.2) due to the stricter conditions applied to select longer tracks for reconstruction.

#### Reconstruction of Laser Events

**Fig. 19:**
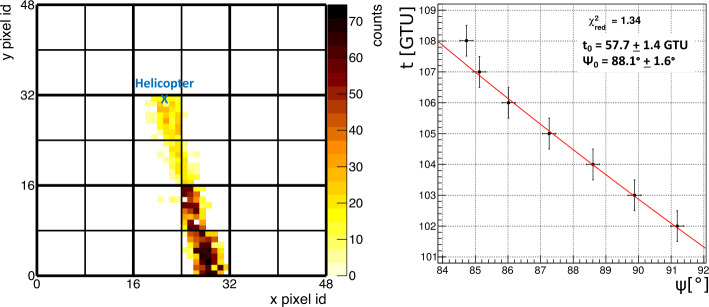
Example laser track observed at 05:40:24 UTC (left panel) with corresponding time profile and fit (right panel). Figure adapted from (Abdellaoui et al. 2018a)

The laser tracks from the underflight were analyzed to reconstruct the laser direction relative to the detector. An illustration of the reconstruction of the geometrical direction of the laser tracks fired from the helicopter using the observables from the balloon is reported in Fig. 18. An example track is shown on the left panel of Fig. 19, with the corresponding two-parameter timing fit on the right panel. In this case, the fit was constrained using the position of the helicopter. If the position of the helicopter is known, the only two parameters that remained to be determined are $t_{0}$ and $\Psi _{0}$ (see Fig. 18). $t_{0}$ indicates the time at which the laser track reaches the closest distance to the detector while $\Psi _{0}$ expresses the angle between the detector’s direction of movement and the line of closest distance between the track and the detector. Similar approach will be used also with a space-based observation. The reflection of the Cherenkov signal on ground or on top of a cloud will provide a similar kind of space-time constrain, as the center of gravity of the Cherenkov bump will allow to identify the pixel where presumably the EAS has landed. In case of a cloud it becomes important to guess it’s altitude. The methodology described in this paper to estimate the CTH will be beneficial also in this regard.

**Fig. 20:**
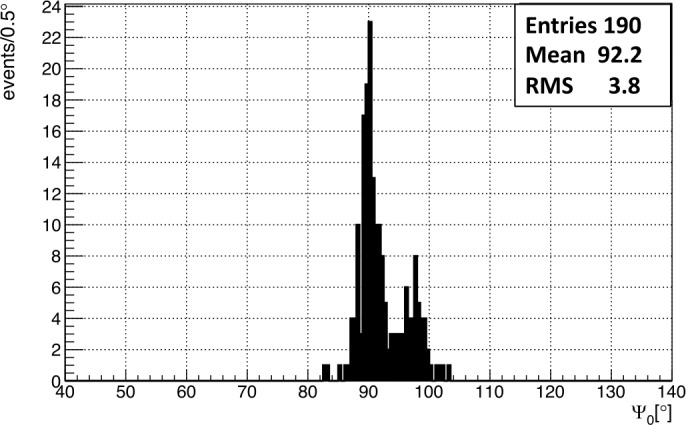
$\Psi _{0}$ angle reconstruction of the helicopter laser shots with the 2-parameter fit method including only tracks with 4 GTUs or more. Figure adapted from (Abdellaoui et al. 2018a)

**Fig. 21:**
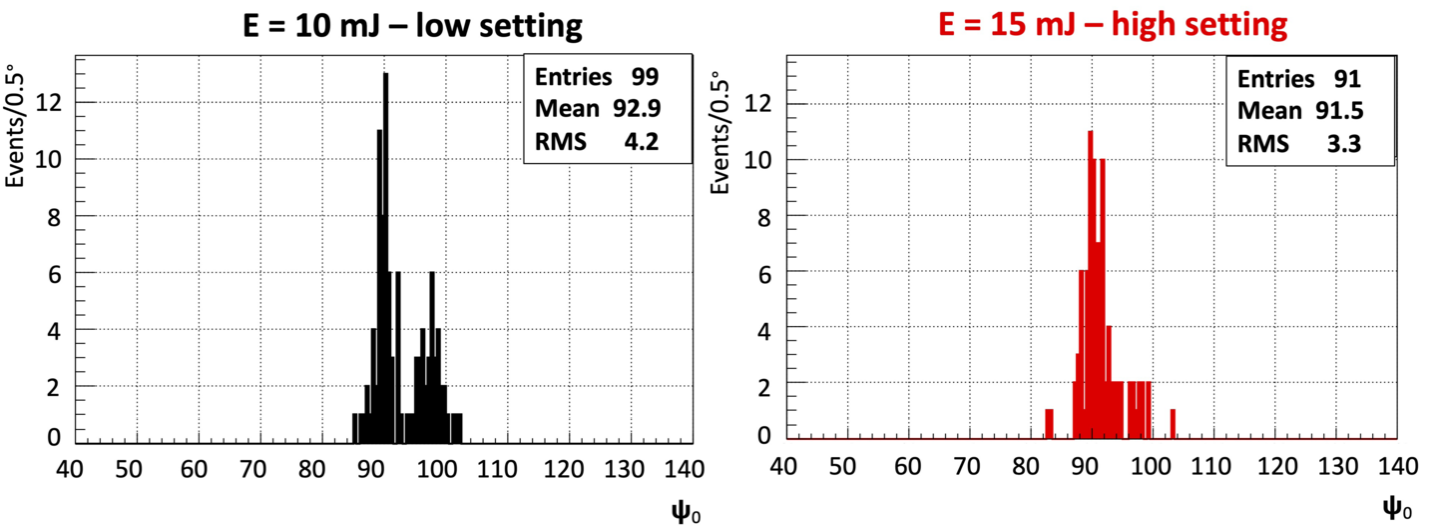
$\Psi _{0}$ angle reconstruction of the helicopter laser shots with the 2-parameter fit method split by laser energy (left: 10 mJ - low setting; right: 15 mJ - high setting). The minimum track duration is 4 GTUs. Figure adapted from (Abdellaoui et al. 2018a)

As shown in Fig. 20 the distribution of the 190 reconstructed events has a mean $\Psi _{0}$ angle of 92.2^∘^ with a standard deviation of 3.8^∘^ which represents the angular resolution. The two visible populations are related to the two energy settings used for the laser. While the lower energy setting is contributing to both populations, the higher setting only contributes to the population centered around 90^∘^. A possible explanation for this behavior is the saturation of pixels inside the track, which shifts the weight of the timing fit. The result of splitting the dataset into high and low energy settings is also shown in Fig. 21.

The expected mean value of the distribution should be slightly above 90^∘^. The reason is that the laser was mounted to produce horizontal tracks (meaning a $\Psi _{0}$ angle of 90^∘^) when the helicopter had a horizontal attitude. However, the helicopter was slightly turned sideways towards the ground by approximately $1^{\circ }$-$2^{\circ }$ to fly a circular pattern. The estimation of the bank angle uses the velocity $v$ of the helicopter and the assumption of truly circular flight pattern ($\theta = \arctan ( v^{2}/(R\cdot g))$) of turning radius $R$. The velocity and position of the helicopter were recorded using the on-board GPS at a 1 Hz rate. Using the position information it was possible to fit circles to the flight pattern and obtain an approximate radius for segments of the flight.

The angular resolution that is obtained is affected by various factors: *EUSO-Balloon* is a prototype instrument designed mainly to demonstrate the *JEM-EUSO* principle. The detector is equipped with only two of the three Fresnel lenses in the original design for the optics, leading to a point spread function of around 9 pixels (∼0.7^∘^). In addition, the PDM has dead spots. The 2.5 μs resolution is too large for a determination of the angular resolution within a few degrees at the short distance between the helicopter and the balloon of 35 km. The angular resolution of 3.8^∘^ does not represent the final resolution of a *JEM-EUSO*-like instrument. The distance between the detector and the shower will be around 10 times larger improving the time resolution issue. Although the laser was too bright to perform an energy reconstruction (pixels were saturated), the beam direction was successfully reconstructed relative to the detector.

### Unidentified Events

**Fig. 22:**
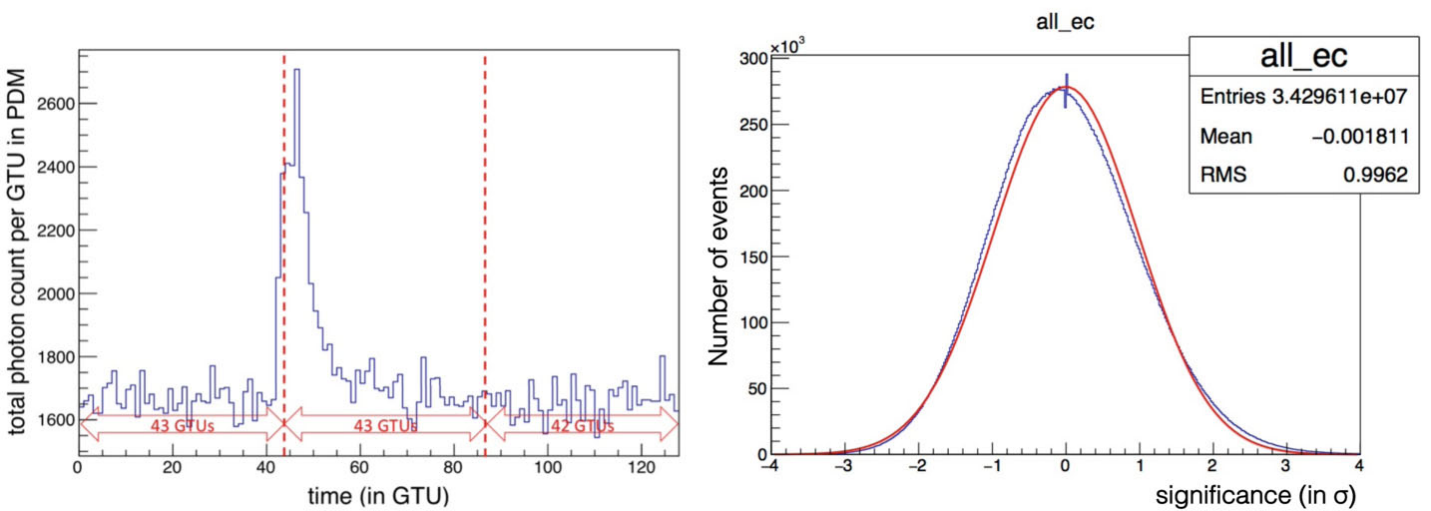
Left: Total photon count of the entire PDM as a function of time (in GTUs) for a specific packet of data. The peak of light in the packet corresponds to the light from a horizontal laser pulse, crossing the FoV. Right: Histogram of the significance of the photon counts in the entire PDM, for all the periods considered without signal (see text) in blue color. A Gaussian fit of the distribution is also shown in red, for comparison. Figure adapted from (Jung 2017)

An analysis was performed to investigate the sensitivity of the instrument to coherent fluctuations of the UV intensity over a large area up to the entire FoV, on timescales of a few to a few tens of μs and search for significant events. The methodology and a detailed discussion of the results is reported in (Jung 2017). While localized signals can be detected at the pixel level, a higher sensitivity to signals with a wider spatial extension can be reached by grouping pixels together, at the MAPMT level, EC level, and PDM level, thereby increasing the signal to noise ratio. In order to determine the significance of these excesses in the count rate, the following procedure is applied. At first, the time interval of each packet is divided into 3 parts, containing respectively 43, 43 and 42 GTUs (see the left panel Fig. 22). The average photon count per GTU during each of these periods, as well as its RMS, $\sigma $ are determined. The smallest average value is kept as indicative of the average UV background during the whole period, and the corresponding RMS is used to determine the significance of the photon count fluctuations. A normalized photon count evolution is then produced by plotting the so-called *significance signal*, defined for GTU number $i$ in packet number $p$, as:

1 $$ \Sigma _{i} = \frac{S_{i} - A_{p}}{\sigma _{p}} $$

where $S_{i}$ is the original signal (photon count) in the PDM at GTU number $i$, and $A_{p}$ and $\sigma _{p}$ are the average and RMS adopted for the packet under consideration. In this way, all signals in all data packets are treated in a consistent way, and signals recorded by different ECs can be compared, even though they do not have the same photon detection efficiencies.

The right panel of Fig. 22 shows the histogram of the significance values, $\Sigma $, of the total photon counts per GTU for the whole PDM. In this histogram, the data are restricted to the periods which served as a reference for the calculation of the average and RMS in the above-described procedure (1/3 of the total data). As a first approximation, one may consider that these periods are free from significant signals (over the considered timescale), and only show statistical fluctuations of the background count. As can be seen, the distribution is reasonably well described by a Gaussian with average 0 and RMS 1, as expected by construction, which supports the assumption that the signal detected in these periods is essentially random background light. It is therefore possible to estimate a spurious event rate based on the selection done to search for signal excesses.

Significant upward fluctuations of the signal were selected by requiring at least N = 3 consecutive GTUs with a photon count more than T sigmas away from the average, the photon count being calculated at EC or PDM level depending on which portion of the FS the significance is calculated. An “event” was defined as a sequence of signals satisfying this criterion. In the following, T = 3 is used as the default value for the significance threshold, but other combinations of N and T values to search for potentially fainter, but still significant signals, were also explored. With the choice N = 3 and T = 3, the probability of a “fake event” (i.e. obtained from a random fluctuation of the background) is easily estimated. Assuming the total photon counts over the entire PDM follow a Gaussian distribution, the probability for the significance signal, $\Sigma $ in one GTU to be larger than 3 is $P_{0} = P(\Sigma \ge 3)= 1.4 \times 10^{-3}$. Therefore, the probability of a fake event (beginning at a given time) is $(P_{0})^{3} = 2.5\times 10^{-9}$. The number of packets recorded during the *EUSO-Balloon* flight and used in this analysis were 259,400. With 128 GTUs each, this amounts to a total of $3.3\times 10^{7}$ GTUs. The probability of finding a fake event in the whole data set is around 8%. If instead, $T = 4$ is chosen for the threshold value, the individual fluctuation probability is $P(\Sigma \ge 3) = 3.2\times 10^{-5}$, which brings the probability of finding one fake event in the whole data set down to a totally negligible value.

Apart from the expected signals produced by the light system of the helicopter flying under *EUSO-Balloon*, the search did not lead to the detection of well identified signals with a possible meteorological or astronomical origin. Nevertheless, a few significant events drew the attention, with different signatures. Three types of signals were identified, which are referred to as: i) “stray light” signals, ii) “mine events” (two of them were found), and iii) “unidentified” (only one event).

The stray light signals are in time coincidence with laser events but were detected in a different part of the focal surface. Therefore, corresponding photons are merely a subsample of the photons scattered by the air molecules along the laser track, reaching the front lens at the same time, but then diffused by the lens system and distributed all over the focal surface, instead of being focused towards the main spot. While this light was sufficient to produce a significant signal in MAPMTs far from the focal point of the incoming light, it corresponds only to a small fraction of the total light received from the laser. This stray light is essentially unnoticeable at the pixel level, and becomes significant only when summed over an entire MAPMT or EC. Therefore, it should not affect the detection and reconstruction of EAS events. This signal is expected as Fresnel lenses, even when perfect, are known to produce secondary structures, in addition to the focal point in the focal surface. Further investigations, including simulations of the optical system as well as experimental studies using the results of laser shot campaigns on the ground, might allow assessing the quantitative effect of these non focused photons as well as other potential imperfections, which could be related to anomalies in the manufacturing of the lenses. However, we stress that the current results, while showing that such secondary “stray light” signals can be observed, also show that they are much weaker than the primary events and should not affect the detection capability of a *JEM-EUSO*-like experiment. It should also be noted that the laser intensity, for which these parasite signals appeared to be significant at the MAPMT level, was much larger than what can be expected for an actual EAS.

**Fig. 23:**
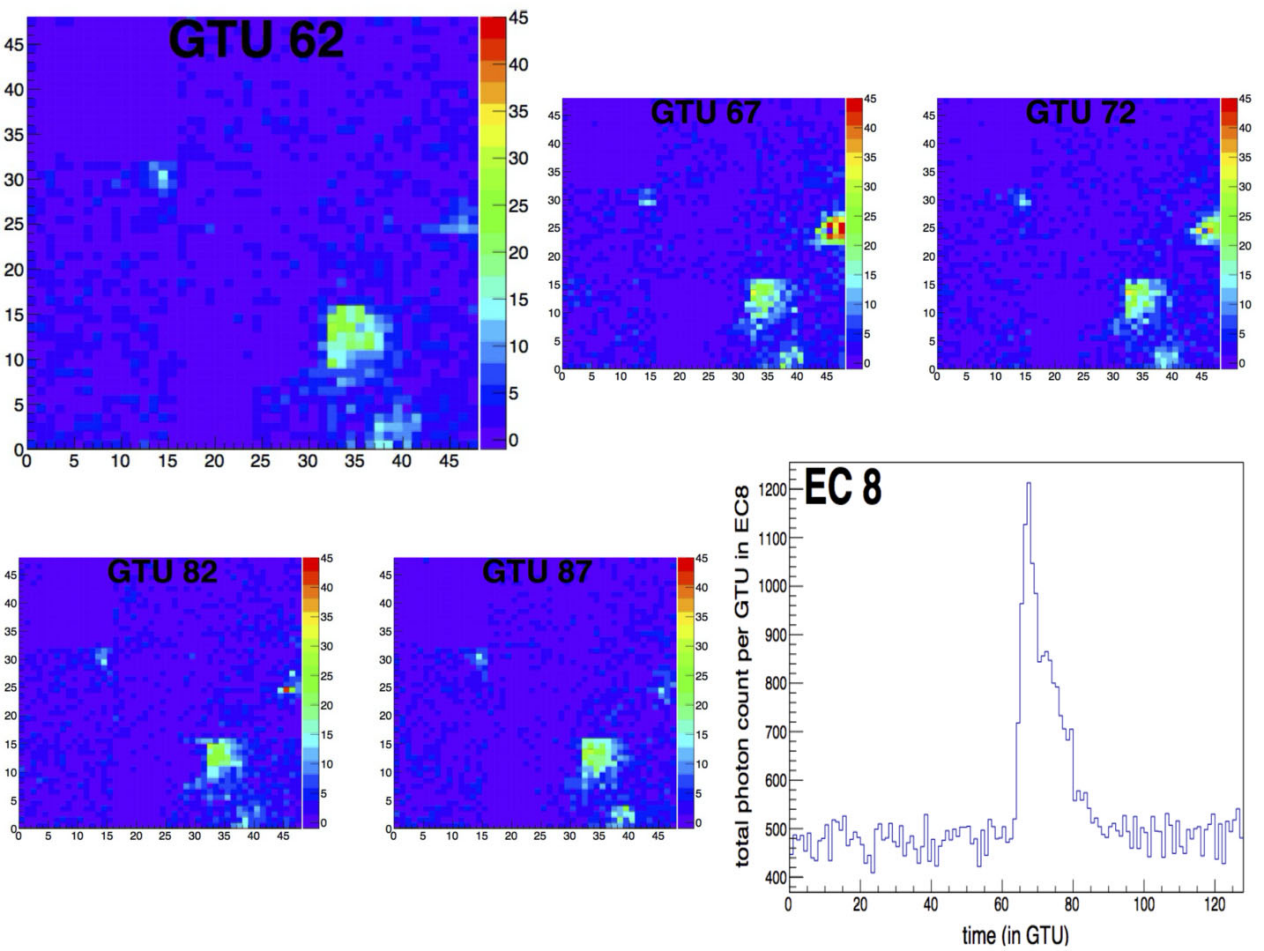
Photon count in each pixel of the entire PDM corresponding to the mine event number 1, at 5 different times indicated by the GTU number within the recorded sequence. The persistent lights are associated with identified mines. The transient event appears at the middle right edge of the FoV, where a mine is also located. The time profile of the transient event is reported in the bottom-right panel. Figure adapted from (Jung 2017)

Two additional interesting events belonging to a different class were detected by the above-mentioned method. They appeared to be associated with human activity as they were both located near mine grounds. Examples of such kind of mine grounds as detected by *EUSO-Balloon* are shown in Fig. 13. Therefore, these events were named “mine events”. One of the two events is reported in the following, as an example. However, the structure of the photon counts on the focal surface look very similar in both events. Figure 23 displays a sequence of images taken by the PDM during the first “mine event”, at 5 different times indicated by the GTU number. The signal has a total duration of 15-20 GTUs, i.e. 40–50 μs, with a sharp rise and high peak, which apparently saturates the dominant pixels, followed by a slower decay, possibly associated with a second peak about 5 GTUs after the first one (see Fig. 23). The time structure and total amount of light appear compatible with a capacitor discharge such as used in a Xenon flasher. The signal indeed appears similar in shape and duration to the one measured with the Xenon flasher carried on the helicopter, and it is known that such type of light sources can be found in industrial facilities.

**Fig. 24:**
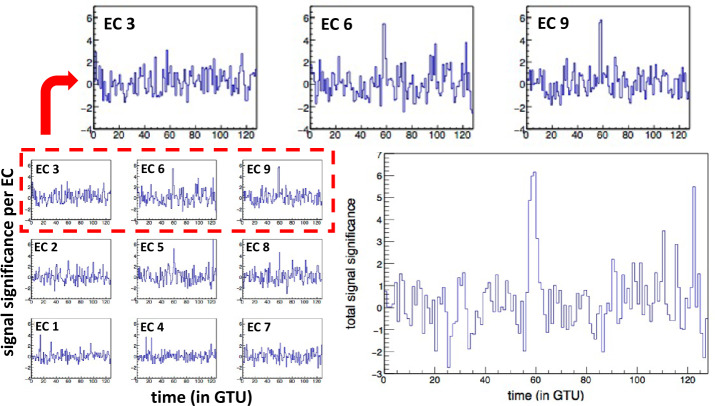
Time sequence showing the photon count significance signal as a function of time (in GTU) in each individual EC (bottom left) and in the whole PDM (bottom right), for the packet in which an unidentified event was recorded. A zoom of three ECs is shown on the top portion of the figure, which is adapted from (Jung 2017)

The most interesting event is the unidentified one shown in Fig. 24, where the significance signal for each individual EC as a function of time (left panel) as well as at the level of the whole PDM (right panel) are plotted. A very clear excess in the photon count rate is visible around the middle of the data packet with a significance of ∼6$\sigma $ at the PDM level. The signal duration is about 4 GTUs, i.e. 10 μs. The fact that it can be visible at least in 4 ECs suggests on the one hand that the signal is not a mere statistical fluctuation of the data count rate (especially since the significance reaches a level of almost 6$\sigma $), and on the other hand that it is indeed not associated with a localised light source in the FoV of the instrument. When looking at individual PMTs, it also appears that most of them show an excess in the photon count during that time, which further suggests a diffuse origin. An excess of light over a large fraction of the focal surface could in principle be due to an event within the instrument itself, for instance associated with the interaction of a cosmic ray in the lenses or other parts of the detector. However, the time scale for such an event would be of the order of a GTU, while a detailed analysis in the 10 GTUs around the excess shows that the significance lasts 3–4 GTUs and moves across the entire FoV. Therefore, the event under consideration is most probably related to an actual excess of incoming light into the optical system. Unfortunately, it looks unlikely that the event could be associated to a UHECR, assuming that most of the diffuse light comes from reflected Cherenkov light over clouds, because the event should have been of energy well above $10^{19}$ eV. Other speculations include a very short and bright light source located outside the FoV of the instrument, e.g. originating from an airplane, whose light could have been reflected off the ground. This uncertainty in discriminating the origin of the event does not pose issues for future balloon missions. A self-trigger, like the one described in Sect. 2, would distinguish these two cases because the airplane signal would show a periodic appearance in the FoV, as measured with Mini-EUSO on ISS.

## Conclusions and Perspectives

The *EUSO-Balloon* flight in 2014 allowed a full scale end-to-end test of most of the key technologies and instrumentation employed in the following projects of the *JEM-EUSO* program. A measurement of the UV intensity in different atmospheric and ground conditions was achieved. The data allowed the development, on real data, of the same methodology that will be used by future missions of the *JEM-EUSO* program in space to determine the exposure. The estimation of the absolute UV intensity proved that the general behavior of the instrument is fairly well understood. The cross-correlation of the UV and IR maps allowed detecting clouds in the UV band. This result is very useful in view of a space-based detector such as *JEM-EUSO* as it will help to select high-quality cosmic ray data. The flight was used as a bench test to verify the possibility to use weather forecasting models in combination with data from an IR camera to determine the cloud top height. The detection of laser events proved the feasibility of the observation of EAS-like events. A detailed analysis of the recorded data searching for significant fluctuations in the detected counts at EC and PDM level allowed the extraction of events with different signatures: i) stray-light events, ii) “mine” events, and iii) one unidentified event which indicate the great variety of signals that could be seen from the top of the atmosphere.

*EUSO-Balloon* was then refurbished for a second flight (*EUSO-SPB1*) of much longer duration (Wiencke and Olinto 2017). The experience obtained in the *EUSO-Balloon* flight turned out to be very useful in planning the *EUSO-SPB1* flight. Moreover, the results of *EUSO-Balloon* are quite complementary to those obtained with *EUSO-SPB1* as this second balloon flew essentially only over the ocean. Both flights provided many insights for the planning of the next balloon mission, *EUSO-SPB2* (Wiencke and Olinto 2019) expected to fly in 2023, as well as the already launched in space *Mini-EUSO* (Bacholle et al. 2021) telescope and the planned large-scale missions *K-EUSO* (Casolino et al. 2017) and *POEMMA* (Olinto et al. 2021).

## List of the Acronyms

ASIC: Application-Specific Integrated Circuit
BaBy: Background Bypass
BT: Brightness Temperature
CCB: Cluster Control Board
CLKB: Clock Board
CNES: Centre National d’Etudes Spatiales, French Space Agency
CPU: Central Processing Unit
CTH: Cloud-Top Height
CTP: Cloud-Top Pressure
CTT: Cloud-Top Temperature
DP: Data Processor
DST: Data STorage
EAS: Extensive Air Shower
EC: Elementary Cell
ECMWF: European Centre for Medium-range Weather Forecasts
ESAF: EUSO Simulation and Analysis Framework
EUSO: Extreme Universe Space Observatory
EUSO-TA: EUSO at Telescope Array
EUSO-SPB1: EUSO Super Pressure Balloon 1
EUSO-SPB2: EUSO Super Pressure Balloon 1
FoV: Field of View
FPGA: Field Programmable Gate Array
GOES: Geostationary Operational Environmental Satellite
GPSR: Global Positioning System Receiver
GTU: Gate Time Unit
HK: HouseKeeping
HVPS: High-Voltage Power Supply
IRAP: Institut de Recherche en Astrophysique et Planétologie
IRcam: InfraRed camera
ISS: International Space Station
JAXA: Japan Aerospace Exploration Agency
JEM-EUSO: Joint Experiments Mission: EUSO
K-EUSO: KLYPVE-EUSO
L1: Front Lens
L2: Diffractive Lens
L3: Rear Lens
LED: Light-Emitting Diode
LVPS: Low-Voltage Power Supply
LWIR: Long-Wavelength InfraRed
MAPMT: Multi-Anode PhotoMultiplier Tube
Mini-EUSO: Multiwavelength Imaging New Instrument for EUSO
MODIS: MODerate Resolution Imaging Spectroradiometer
NASA: National Aeronautics and Space Administration
NOAA: National Oceanic and Atmospheric Administration
NWP: Numerical Weather Prediction
PAO: Pierre Auger Observatory
PCE: Photon Collection Efficiency
PDM: Photo-Detector Module
PMMA: Polymethyl-methacrylate
POEMMA: Probe Of Extreme Multi-Messenger Astrophysics
PPS: Pulse Per Second
PSF: Point Spread Function
RAID: Redundant Array of Independent Disks
RMSE: Root Mean Square Error
SPACIROC: Spatial Photomultiplier Array Counting and Integrating ReadOut Chip
SSD: Solid State Disk
SWIR: Short-Wavelength InfraRed
TA: Telescope Array
UHECR: Ultra-High Energy Cosmic Ray
UTC: Coordinated Universal Time
UV: UltraViolet
WRF: Weather Research and Forecasting
